# Intermittent muscle activity in the feedback loop of postural control system during natural quiet standing

**DOI:** 10.1038/s41598-017-10015-8

**Published:** 2017-09-06

**Authors:** Hiroko Tanabe, Keisuke Fujii, Motoki Kouzaki

**Affiliations:** 1 Graduate School of Arts and Sciences, The University of Tokyo, 3-8-1 Komaba, Meguro, Tokyo, 153-8902 Japan; 2 Center for Advanced Intelligence Project, Institute of Physical and Chemical Research, 6-2-3 Furuedai, Suita Osaka, 565-0874 Japan; 3 Graduate School of Human and Environmental Studies, Kyoto University, Yoshida-nihonmatsu, Sakyo-ku, Kyoto, 606-8501 Japan

## Abstract

The origin of continual body oscillation during quiet standing is a neural-muscular-skeletal closed feedback loop system that includes insufficient joint stiffness and a time delay. Thus, muscle activity and joint oscillations are nonlinear during quiet standing, making it difficult to demonstrate the muscular-skeletal relationship experimentally. Here we experimentally revealed this relationship using intermittent control theory, in which non-actuation works to stabilize the skeletal system towards equilibrium. We found that leg muscles were activated/inactivated when the state point was located in the opposite/same direction as the direction of anatomical action, which was associated with joint torque actuating the body towards equilibrium. The derivative values of stability index defined in the phase space approximately 200 ms before muscle inactivation were also larger than those before activation for some muscles. These results indicate that bipedal standing might be achieved by monitoring the rate of change of stability/instability components and generating joint torque to stabilize the body. In conclusion, muscles are likely to activate in an event-driven manner during quiet standing and a possible metric for on/off switching is SI dot, and our methodology of EMG processing could allows us to extract such event-driven intermittent muscle activities.

## Introduction

Efficient balance recovery and fall prevention against postural perturbation coming from dynamically changing environment is crucial for everyone. In the elderly people, poor postural balance leads future falls^1, 2^ and difficulties in recovering from small postural perturbations^3^. Aging also alters multi-link joint coordination during standing^4^, indicating the importance of investigating how we control multiple joints simultaneously and coordinatively. Given its multiple joints, the human body naturally sways in a non-linear and non-stationary manner during quiet standing^5–14^, which is due to a closed-loop system of human postural control. The central nervous system (CNS) generates motor commands based on the integrated sensory cues^15^ of body fluctuations following which muscle activities occur so as to maintain an upright posture. It has been difficult to investigate the relationship between joint oscillation and muscle activities during quiet standing because muscle activities are a mixture of the results of and reasons for joint oscillations and because the feedback loop contains a time lag, which takes for sensory feedback, neural processing, and joint actuation. However, it is necessary to determine the relationship among joint oscillations, muscle activity, and joint torque output experimentally in order to understand the mechanism of postural control.

Mechanically, passive stiffness caused by joint viscoelasticity of the muscle-tendon-ligament is insufficient to overcome the gravitational toppling torque during standing^16, 17^. Intermittent feedback control strategy has recently been proposed as a control model of such unstable body plant during standing; this control strategy model assumes that a closed-loop feedback control occurs intermittently (thus, there is a switching mechanism) for the stabilization of the system. Although many studies deal with stick balancing, recent modeling studies suggest that similar ideas used in modeling studies likely apply to postural control during quiet standing^18^. Intermittent feedback control strategy is roughly divided into two types: clock-driven and event-driven model. The clock-driven type, with which switching of feedback control (presence/absence of the feedback) occurs at regular intervals, was developed by P. Gawthrop and his colleagues^19, 20^ and is favored by a group of I.D. Loram^21^. The event-driven type, on the other hand, are divided into further three types: 1) a model in which the switching threshold does not depend on the time delay of the system^22^, 2) a model assuming that the flow in the phase space of actual kinematics data is imitated by a flow associated with a saddle point of a dynamical system without time delay^23–26^, and 3) a model assuming that intermittent feedback control is required for postural control because the control is tuned near an edge of stability or there is a sensory dead zone present^27–30^. All of these studies have shown that human postural control mechanism is likely to be described by the intermittent feedback control, but its switching mechanism “clock-driven or event-driven” is still controversial. The reason for the difficulty in experimentally validating the switching mechanism taken in the intermittent control theory is that muscle activities repeat small activation and inactivation in a non-stationary manner and thus, it is difficult to detect such on/off activity from electromyography (EMG) signals.

In this study, we hypothesized that intermittent muscle activation and inactivation occur in evend-driven manner based on joint angle and velocity and generate joint torque to stabilize each body segment (as we have proposed in our previous study^31^). Detecting “on-periods” and “off-periods” of muscle activities might be useful for the investigation of the kinetic-kinematic relationship by looking at the location of the state point (joint angle and velocity information) during each period. Muscle activity is a mixed outcome of impedance control, postural reflex, and active feedback control. In this study, we consider phasic muscle on/off activity to be due to the feedback loop via the basal ganglia, prefrontal cortex, and premotor cortex and to provide low-bandwidth feedback at longer frequencies^32–34^. Nomura *et al*.^35^ have reported the way to decompose EMG signals from soleus muscle during quiet standing into tonic (which involves muscle spindle and Golgi tendon organ feedback and provides tonic equilibrium joint moments via tonic stretch reflexes^36^) and phasic components by using low-pass filtering of two kinds of cutoff frequencies. However, the actual human body has a multi-link structure, which is a non-integrable system, making it difficult to reveal the function of intermittent activities in multiple muscles during natural standing. For experimentally validating event-driven intermittent feedback control as a human postural control mechanism, we first aimed to investigate the input-output relationship (that is, the relationship between muscle activity and joint oscillations) by statistically comparing joint fluctuations or torque output between muscle on- and off- periods. Then we experimentally compared the stability of the system between on- and off- periods within the event-driven control loop for each muscle using a following steps; we first created a control model and actuate the pendulum based on it, and then we compared simulated and experimental data for showing that the control model was physiologically reasonable (this was based on our previous work^31^), which allowed us to investigate whether the actual muscle on/off activity occur based on the same control mechanism as what was used for actuating the pendulum in simulation. If there exists a difference in the stability (as a triggering event) between on- and off- periods, it would help provide an evidence for event-driven intermittent feedback control for postural maintenance.

The objectives of this study were 1) to experimentally determine the direct relationship among joint fluctuations, muscle activity, and joint torque output, by extracting phasic muscle activity from EMG data, and 2) to investigate whether the joint sway dynamics based on experimentally extracted muscle on/off activity matches the event-driven intermittent feedback control theory by comparing joint oscillations between simulation and experimental data during on- and off- periods. To the best of our knowledge, this is the first study to combine experimental and computational methodologies to deepen our understanding of the mechanism of postural control of naturally oscillating human bipedal standing.

## Results

### On/off centers in the phase and torque planes

**Figure 1:**
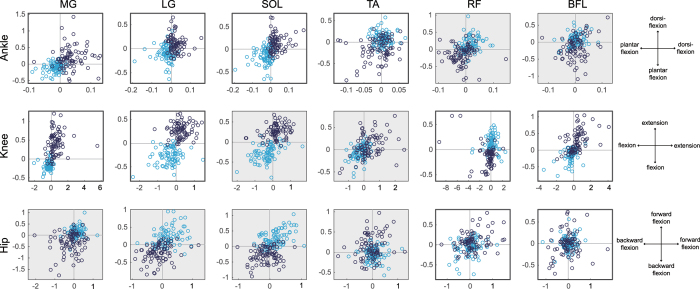
On/off centers in the phase planes of the ankle (top), knee (middle), and hip (bottom). Vertical and horizontal axes represent angular position and velocity, respectively; positive sign represents ankle dorsiflexion, knee extension, and hip anteflexion. Dark blue and light blue circles represent the centers of on-area and off-area, respectively, for all trials (80 samples for each muscle). Gray background represents an anatomically indirect joint-muscle relationship (e.g., MG cannot actuate the hip directly).

**Figure 2:**
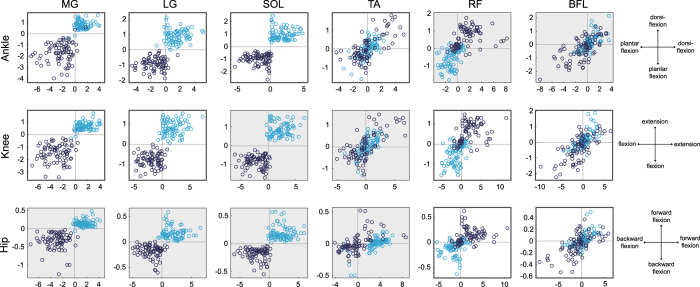
On/off centers in the torque planes of the ankle (top), knee (middle), and hip (bottom). Vertical and horizontal axes represent joint torque and joint torque velocity, respectively; positive sign represents ankle dorsiflexion, knee extension, and hip anteflexion. Dark blue and light blue circles represent the centers of on-area and off-area, respectively, for all trials (80 samples for each muscle). Gray background represents an anatomically indirect joint-muscle relationship.

We measured EMG signals from six muscles of both legs during natural quiet standing to detect on-periods (activation) and off-periods (inactivation) of each muscle. We then calculated the center of the mixed Gaussian distribution for each on/off area in the phase planes and torque planes, which are related to on/off trigger timing and control output, respectively (for details, see Method section). Figures 1 and 2 show 80 samples (from 8 subjects undergoing 5 trials on both legs) of on/off centers for each muscle in the phase planes and torque planes, respectively, of the ankle (top), knee (middle), and hip (bottom). Red and blue circles represent on and off centers, respectively. Most of the muscles (especially triceps surae muscles) were activated (on-area) and inactivated (off-area) when the state point was located in the opposite direction and in the direction of anatomical action, respectively (Fig. 1). The results of one-sample t-test on five-trial data for each participant showed statistically significant divergence of on/off centers from the x-axis and the y-axis. For example, the values of the horizontal axis on the ankle phase plane (ankle angle) for the center of the on-period of participant 1 significantly diverged from the vertical axis (|t_4_| = 3.87, p < 0.05). This indicates that intermittent muscle activities occur based on the location of the state point in the phase planes. All of the results of the t-test for all joints and muscles are shown in Supplementary Material B.

In addition, the intermittent muscle on/off activities generate joint torques that is in the direction of anatomical action for each muscle (Fig. 2). In particular, on/off areas of triceps surae muscles were explicitly distributed over the third and first quadrants of the ankle and knee torque planes, suggesting that anti-gravity muscles are intermittently activated in order to deal appropriately with the ever-present gravitational toppling torque during quiet standing. The results of one-sample t-test on five-trial data for each participant showed statistically significant divergence of on/off centers from the x-axis and the y-axis. For example, the values of the horizontal axis on the ankle torque plane (ankle torque) for the center of the on-period of participant 1 were significantly diverged from the vertical axis (|t_4_| = 12.0, p < 0.05). This indicates that intermittent muscle activities generate statistically non-zero joint torque that takes joints back to the equilibrium (i.e., baseline). All of the results of the t-test for all joints and muscles are shown in Supplementary Material B.

### Stability index (SI)

**Figure 3:**
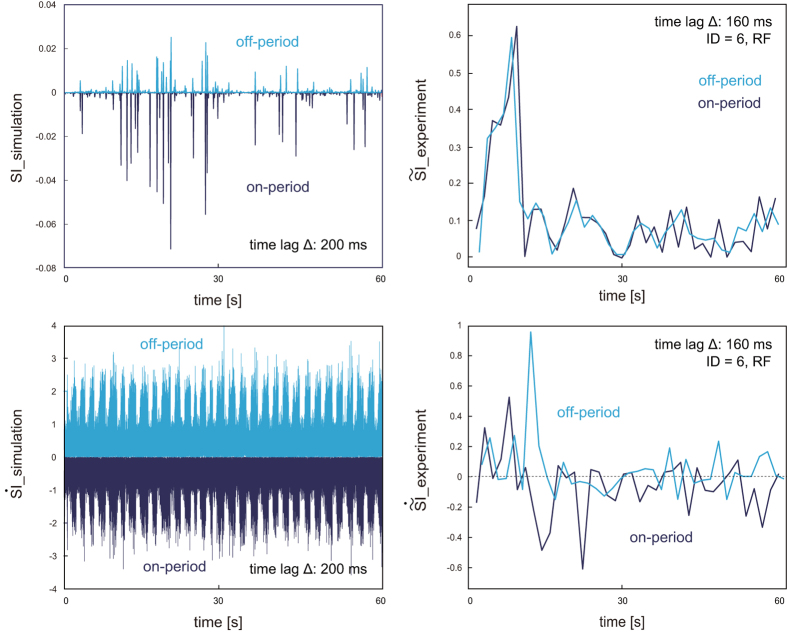
Examples of stability index (SI) and its derivative ($\tilde{S}I$) for simulation (left) and experimental (right) data. Vertical axis represents averaged SI or $\tilde{S}I$ for each on (dark blue) and off (light blue) period. Left: SI (top) and $\tilde{S}I$ (bottom) 200 ms before each on/off period for simulation data. Right: SI (top) and $\tilde{S}I$ (bottom) 160 ms before each on/off period of rectus femoris (RF) of one participant (ID = 6).

We implemented a computer simulation with a triple inverted pendulum that was controlled in accordance with and event-driven intermittent feedback control theory^31, 37, 38^. We defined the stability index (*SI*) by using the stable and unstable components calculated in the phase space. Then, we investigated the actual mechanism of postural control inside the CNS by comparing *SI* during on- and off- periods between simulation and experimental data. This was performed in order to verify the following hypothesis of the intermittent feedback control theory: intermittent on/off switching is triggered based on the ratio of stability/instability components. Figure 3 shows examples of SI for simulation (left-top) and $\tilde{S}I$ for experimental data (right-top), shown as averaged values for each on/off period during one 120-s trial. For simulation data, *SI* was greater/less than zero 200 ms before each off/on period, respectively, because on/off switching was implemented based on this SI value in eq. (4). However, there was no significant difference in $\tilde{S}I$ with any time lag ranging from 0 to 400 ms for all muscles of all participants. Thus, we re-implemented simulation with intermittent control again based on eq. (7) and found $\tilde{S}I$ to be greater/less than zero 200 ms before each off/on period, respectively (Fig. 3 left-bottom). For experimental data, the derivative value of the stability index during off-periods for one piece of experimental data ($\tilde{S}I$ for participant 6’s RF on/off switching; Fig. 3 right) was also greater than those of on-periods 160 ms before the switching.

**Figure 4:**
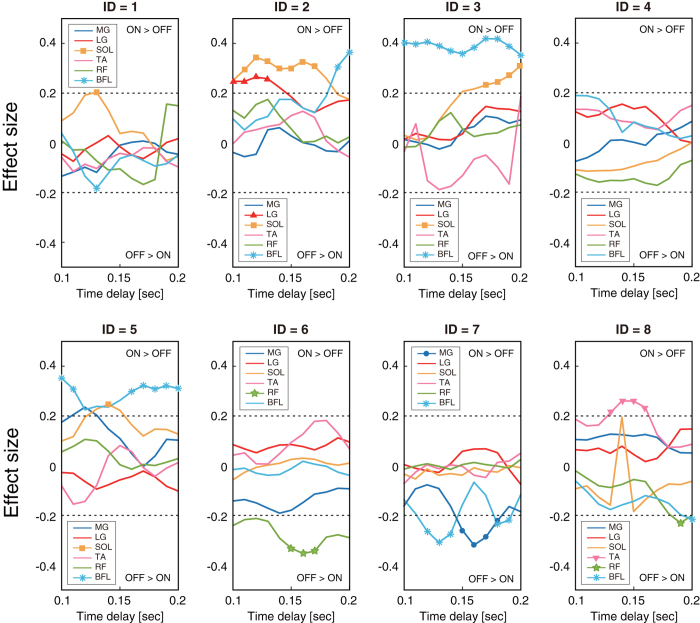
The effect sizes for the comparison of $\tilde{S}I$ between on and off periods with a time lag ranging from 0.1 to 0.2 s for all muscles of every participant. Negative value of effect size represents the larger $\tilde{S}I$ during off-periods compared with that in on-periods, which accords with the intermittent control theory. Horizontal axis represents time lag and vertical axis represents effect size. Marks on each line (●, ▲, ■, ▼, ★, and *□ are for MG, LG, SOL, TA, RF, and BFL, respectively) show significant differences between on- and off- periods. Horizontal dashed lines show the absolute value of the effect size of 0.2, which we consider to be a statistically meaningful value.

We then compared $\tilde{S}I$ between on and off periods with a variety of time lags ranging from 100 to 200 ms in order to investigate the possibility that actual muscle activation and inactivation occur based on the derivative of SI within a range of physiologically reasonable time lag. Figure 4 shows the effect size for the comparison in $\tilde{S}I$ between on and off periods for all the muscles of every participant. The negative value of effect size represents the larger $\tilde{S}I$ during off-periods compared with on-periods, which accords with the intermittent control theory. Some muscles of participants 1 (BFL), 6 (RF), 7 (MG and BFL), and 8 (RF) showed statistically larger $\tilde{S}I$ during off-periods than during on-periods with a time lag rangin from 100 to 200 ms. On the other hand, participants 1 (SOL), 2 (LG, SOL, BFL), 3 (SOL, BFL), 5 (BFL), and 8 (TA) showed larger $\tilde{S}I$ during on-periods, which contradicts the intermittent control theory.

## Discussion

### Kinetic-kinematic relationship within the postural control system

The first objective in this study was to experimentally determine the direct relationship among phasic muscle activity, joint torque output, and joint oscillations during quiet standing. The EMG magnitude and fluctuation are very small during quiet standing, which makes it difficult to observe agonist-antagonist muscle activation patterns just by evaluating its amplitude or variability. Also, some muscles such as soleus show tonic activity during quiet standing, so it has been impossible to detect on/off switching for such muscles. Under these situations, muscle activation patterns during standing have mainly been investigated when participants respond to postural perturbations, which induce much larger body sway and muscle activities (e.g. in the work of Horak *et al*.^39^). On the other hand, our muscle on/off discrimination method is not affected by the magnitude and variability of the signals because we filtered the signals by using a cutoff frequency based on task-specific CoP fluctuation for making a trend curve. Thus, this discrimination method could be applicable to other postural tasks for investigating muscle activation patterns by changing the cutoff frequency depending on experimental tasks or participants.

Most of the muscles were activated (on-area) and inactivated (off-area) when the state point was located in the opposite direction and in the direction of anatomical action, respectively (Fig. 1). This result corresponds to our hypothesis of an event-driven intermittent feedback control strategy, in which the control input (i.e., muscle activations via the CNS) is triggered based on the location of the state point in the phase plane. That is, our results indicate that phasic muscle activities during quiet standing are triggered based on such a mechanism that is described as dynamics in the phase space of saddle type. This tendency is particularly pronounced in the ankle and knee phase planes (Fig. 1, top and middle). On/off area separation in the hip phase plane was much more obvious for the anatomically irrelevant triceps surae muscles, but not for thigh muscles (Fig. 1, bottom). This may be because shank muscles that act to counter the force due to gravity play important roles for postural balancing and their activities indirectly affect the fluctuations of the hip joint via the skeletal transmission of force. Activations of the other muscles around the hip (e.g. gluteus maximus or psoas muscles) could affect the hip oscillations rather than thigh muscles, however, we could not obtain clear EMG data of gluteus maximus and major psoas (the signals were small relative to the noise). The role of gluteal and trunk muscles for the control of the hip during standing would be the next step to be clarified.

**Figure 5:**
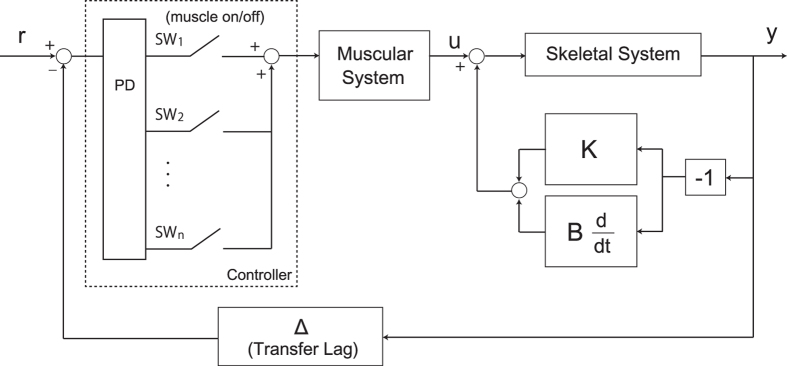
Block diagram of event-driven intermittent feedback control of human quiet standing. On/off switching (SW_k_) of each muscle (or each group of muscles) is triggered depending on the error between current state y (consisting of angular displacements and velocities) and reference value r. The reference value of r could be set for each muscle or each group of muscles. The intermittent muscle activities generate joint torque and, together with passive torque (stiffness and damping components K and B) without a time delay, control input u to actuate the skeletal system and induce joint fluctuations.

Figure 5 shows the postural control model with intermittent feedback control via phasic muscle activity. Open/close of the switches (SW_1_, SW_2_, …, SW_n_) in Fig. 5 represent the absence/presence of the descending control command from the cerebral cortex, which actuates each muscle or muscle modules. Because we found some pairs of muscles did not show high on/off consistency (Table 1), we do not assume that all the switches are either open or closed at the same time. It could be possible for some switches remain open at all time because of the presence of sensory dead zone^40^ and the fact that only the requisite muscles are actuated during standing^41^. However, we do not assume that some switches remain closed all the time because in our postural control model, we take up muscle activation/inactivation associated with postural control and we do not consider continuous muscle activation (continuous switch closure) to contribute to event-related postural stabilization. We assume that such continuous muscle activation affect joint viscoelasticity as a passive contribution to postural maintenance. Overall, we assume in Fig. 5 that different switches (for each muscle or each muscle module) have different threshold. Our experimental data partly suggested that the CNS generates control input as an intermittent switching for each group of muscles or each muscle based on the feedback information of joint oscillations. A conceivable muscle group could be consisted of triceps surae muscles because their on/off consistencies were more than 70% for every participant (Table 1). The limitation of this study is that we investigated the independent contribution of each muscle activity to the postural control. Because the contribution of muscle activities should be related to each other, refining the framework of switching mechanism for postural control is necessary for the future work.

**Table 1:** On/off consistency between different muscles.

	**Right**						**Left**					
	MG	LG	SOL	TA	RF	BFL	MG	LG	SOL	TA	RF	BFL
**On/off consistency of participant 1**. Consistency among triceps surae muscles were high both in right and left leg. Right RF showed relatively high consistency with right TA and BFL. Left RF showed relatively high and low consistency with TA and SOL, respectively. There was no on/off consistency between left and right leg muscles except for TA.												
**Right**	MG	72.5(5.8)	79.6(4.3)	50.7(12.2)	45.1(12.8)	47.3(11.6)	54.3(5.1)	54.9(4.7)	52.1(4.3)	51.4(5.1)	44.2(5.6)	59.0(6.4)
		LG	70.6(5.9)	52.9(7.0)	46.6(11.2)	46.3(9.3)	50.0(4.7)	51.8(5.2)	49.2(1.5)	50.2(5.3)	48.6(6.1)	56.4(6.1)
			SOL	41.3(12.2)	40.1(13.6)	46.0(11.7)	51.7(5.5)	53.1(4.6)	53.3(4.1)	44.3(4.1)	42.4(4.3)	56.1(4.2)
				TA	61.2(9.6)	57.3(5.0)	52.4(4.2)	49.9(5.2)	47.3(4.8)	67.3(4.0)	54.9(5.6)	54.7(4.1)
					RF	61.1(7.1)	53.5(7.4)	47.9(4.3)	48.9(8.5)	57.2(6.1)	57.6(5.0)	50.2(6.3)
						BFL	55.1(4.4)	50.6(4.5)	51.1(3.7)	53.3(5.9)	52.2(2.9)	53.8(6.0)
**Left**							MG	74.0(3.8)	81.6(5.8)	52.1(6.3)	41.7(5.2)	59.1(3.7)
								LG	75.6(6.4)	47.6(1.2)	41.7(4.0)	60.0(1.4)
									SOL	43.4(5.8)	37.5(4.1)	56.6(4.8)
										TA	60.1(4.6)	54.1(4.0)
											RF	53.5(5.7)
												BFL
**On/off consistency of participant 2**. Consistency among triceps surae muscles were high both in right and left leg. Left RF showed relatively low consistency with left LG and SOL. Consistency between left MG and BFL was also high. Some muscles showed high on/off consistency with contralateral leg muscles; MG, TA, and BFL between both legs, and right MG and left BFL. Also, there were some muscles that showed low consistency with contralateral leg muscles; right TA and left LG and SOL, and right RF and left LG and SOL.												
**Right**	MG	71.3(6.5)	67.0(4.7)	54.9(8.0)	48.5(10.8)	58.6(8.5)	63.5(11.9)	52.7(5.4)	47.0(9.0)	54.0(3.9)	49.5(8.9)	64.0(5.9)
		LG	75.7(7.1)	50.4(8.9)	48.5(8.8)	52.1(4.5)	53.6(3.8)	51.4(4.5)	51.2(8.9)	48.0(4.7)	50.4(6.2)	57.2(5.9)
			SOL	41.9(5.8)	40.1(8.8)	55.9(5.1)	57.5(2.7)	58.1(4.5)	58.4(8.4)	47.1(5.0)	42.4(5.6)	57.9(7.5)
				TA	58.5(6.8)	47.6(6.5)	47.5(10.0)	39.3(3.9)	37.8(4.3)	61.3(7.8)	58.1(4.1)	55.0(5.0)
					RF	48.3(5.8)	44.2(4.5)	38.2(5.2)	38.1(3.5)	52.2(4.7)	59.5(4.0)	51.0(3.3)
						BFL	70.9(7.7)	57.4(4.4)	56.3(5.5)	53.3(8.9)	48.1(6.0)	63.0(6.2)
**Left**							MG	66.3(3.8)	63.2(6.0)	56.3(6.2)	41.6(4.7)	63.5(7.1)
								LG	75.5(4.0)	54.6(5.9)	39.7(4.9)	49.9(4.2)
									SOL	48.7(6.7)	38.8(4.9)	45.3(3.8)
										TA	52.1(2.8)	55.3(2.8)
											RF	55.5(4.5)
												BFL
**On/off consistency of participant 3**. Consistency among triceps surae muscles were high both in right and left leg. Left TA showed high on/off consistency with left triceps surae muscles, and right TA showed high consistency with right MG and BFL. Also, activation and inactivation of MG and BFL were consistent for both legs. RF (especially of right leg) activation/inactivation was opposite to those of triceps surae muscles of both right and left leg. High consistency in the same muscle between both legs was observed in MG, RF, and BFL.												
**Right**	MG	71.1(2.2)	69.0(4.5)	67.0(6.1)	35.0(4.0)	62.3(8.5)	69.0(5.1)	57.1(5.7)	56.4(5.5)	58.6(3.3)	41.9(6.2)	62.8(13.7)
		LG	79.1(5.9)	59.9(7.5)	32.8(10.1)	50.1(3.7)	58.2(5.9)	55.1(7.9)	55.9(8.2)	50.3(4.9)	37.9(6.1)	52.6(8.2)
			SOL	58.1(6.7)	30.9(12.7)	47.4(3.5)	58.1(5.4)	55.7(7.2)	55.6(10.5)	48.6(5.7)	36.1(6.4)	51.1(7.9)
				TA	51.1(2.5)	62.2(4.8)	53.8(5.9)	46.9(6.3)	46.3(7.3)	52.7(7.9)	47.1(4.9)	54.8(10.1)
					RF	58.8(6.2)	36.0(2.2)	38.2(4.6)	35.4(6.0)	44.2(8.9)	62.8(8.2)	49.9(6.2)
						BFL	58.7(8.5)	49.0(6.7)	48.3(5.3)	55.2(8.8)	54.7(4.3)	66.5(9.1)
**Left**							MG	72.1(5.1)	69.0(6.3)	65.2(4.3)	41.8(3.8)	64.0(12.3)
								LG	74.6(6.2)	64.3(5.1)	41.3(2.3)	50.5(7.5)
									SOL	60.5(8.1)	39.9(3.5)	46.7(8.2)
										TA	48.0(5.7)	55.0(8.0)
											RF	55.2(8.9)
												BFL
**On/off consistency of participant 4**. Consistency among triceps surae muscles were high both in right and left leg. Left RF showed low consistency with left LG and SOL. Also, left MG showed high consistency with left TA and BFL, and right TA showed high consisntency with right BFL. For contralateral consistency, right TA showed high consistency with left MG, TA, and BFL, and right BFL showed consistency with all of the left muscles. On/off of right MG and left BFL was also consistent.												
**Right**	MG	79.5(4.6)	76.1(5.1)	55.6(12.2)	47.7(9.9)	53.8(5.1)	53.8(3.5)	56.7(6.6)	56.2(6.6)	46.4(6.2)	45.0(4.7)	62.3(4.9)
		LG	71.2(5.3)	57.8(7.5)	47.2(9.9)	54.5(5.2)	52.9(2.6)	57.0(9.4)	57.4(8.1)	47.5(7.2)	45.0(3.7)	59.2(3.5)
			SOL	53.7(8.0)	47.3(7.5)	54.6(6.0)	53.2(3.5)	53.8(4.4)	53.0(4.4)	44.0(5.3)	45.4(8.3)	59.0(6.3)
				TA	54.2(4.5)	65.2(7.1)	63.0(6.9)	54.9(10.4)	54.4(9.4)	67.3(8.8)	48.5(6.8)	64.6(9.8)
					RF	54.3(2.7)	50.4(1.8)	50.8(11.2)	48.9(10.0)	56.1(5.6)	51.3(5.8)	48.0(3.0)
						BFL	65.0(7.3)	66.3(11.6)	66.5(11.3)	65.5(7.5)	37.7(10.7)	64.0(10.0)
**Left**							MG	66.4(6.5)	65.8(7.1)	63.8(7.9)	43.9(8.2)	65.3(9.0)
								LG	88.2(4.8)	54.8(12.3)	27.3(3.0)	54.4(7.0)
									SOL	52.9(11.0)	24.8(4.9)	54.9(7.4)
										TA	48.3(9.6)	59.2(10.5)
											RF	53.5(12.0)
												BFL
**On/off consistency of participant 5**. Consistency among triceps surae muscles were high both in right and left leg. Right BFL showed high consistency with right MG and TA, and left BFL showed high consistency with left RF. Also, left LG on/off was oppositely consistent with that of left RF and BFL. Contralateral consistency was observed between TA and MG, as well as between left MG and right BFL.												
**Right**	MG	65.8(3.1)	62.8(4.5)	53.3(7.0)	50.5(5.1)	67.7(5.6)	59.6(7.4)	52.5(2.2)	50.0(5.4)	69.3(3.2)	43.9(6.8)	51.8(8.5)
		LG	70.9(5.0)	54.2(4.1)	42.7(10.2)	56.3(4.1)	51.2(3.4)	48.3(4.5)	50.1(7.2)	56.2(4.5)	48.2(3.7)	48.4(3.3)
			SOL	51.2(5.4)	43.2(10.2)	50.8(3.8)	46.3(3.0)	44.9(4.9)	46.9(6.9)	58.4(4.9)	47.6(4.0)	50.2(3.7)
				TA	49.9(7.5)	66.7(4.2)	63.9(6.6)	52.4(5.6)	52.9(5.1)	47.1(7.9)	47.5(4.8)	46.7(4.1)
					RF	51.0(6.7)	48.5(6.8)	47.4(5.3)	46.4(3.4)	50.2(4.4)	47.0(10.2)	54.3(3.7)
						BFL	73.7(3.4)	57.5(3.4)	56.3(1.8)	58.4(6.9)	47.3(6.5)	47.1(7.9)
**Left**							MG	66.8(4.7)	67.3(3.3)	48.9(5.3)	51.6(3.7)	47.0(12.3)
								LG	75.4(10.6)	47.8(3.0)	36.5(8.2)	37.1(8.8)
									SOL	43.9(5.6)	42.5(10.6)	40.3(8.6)
										TA	49.8(7.3)	54.5(5.4)
											RF	60.9(7.4)
												BFL
**On/off consistency of participant 6**. Consistency among triceps surae muscles were high both in right and left leg. Ipsilateral consistency was observed between MG and TA and between TA and RF in both legs. Right BFL on/off was consistent with that of right LG and SOL, and left BFL on/off was consistent with that of left MG, TA, and RF. Also, right SOL and RF showed opposite sonsistency. Contralateral consistency was observed in MG, SOL, TA, RF between both legs, and between MG and BFL, between LG and SOL, and between TA and RF. Right BFL on/off was consistent with that of left LG and SOL, and left BFL on/off was consistent with that of right TA.												
**Right**	MG	67.4(5.1)	69.7(6.7)	63.5(8.0)	52.3(2.0)	59.1(7.4)	62.3(8.2)	57.2(3.6)	61.1(2.9)	51.7(4.9)	53.3(2.0)	66.6(3.7)
		LG	82.4(4.2)	53.0(5.5)	40.3(5.5)	61.6(3.9)	56.7(3.9)	59.9(5.3)	65.1(4.4)	45.8(2.1)	46.7(8.6)	54.6(3.7)
			SOL	48.4(4.7)	39.5(6.1)	60.5(5.4)	56.3(5.6)	60.5(6.6)	63.4(3.5)	44.9(0.6)	43.7(6.6)	55.8(4.1)
				TA	69.7(7.1)	45.7(5.5)	58.1(6.7)	51.8(7.9)	53.5(6.1)	67.8(6.3)	68.0(5.1)	66.6(7.6)
					RF	46.1(5.8)	55.5(5.1)	48.6(8.5)	47.4(8.9)	66.1(11.8)	68.3(11.7)	58.5(5.5)
						BFL	67.6(2.4)	63.3(3.0)	63.5(4.7)	49.4(8.0)	45.3(4.9)	54.0(4.9)
**Left**							MG	68.3(4.2)	67.1(2.7)	64.4(4.9)	53.2(5.3)	66.9(6.4)
								LG	77.5(5.8)	58.6(7.6)	44.3(12.2)	59.5(7.0)
									SOL	52.8(8.2)	44.6(9.9)	59.9(4.9)
										TA	64.2(9.0)	61.5(4.8)
											RF	62.4(5.9)
												BFL
**On/off consistency of participant 7**. Consistency among triceps surae muscles were high both in right and left leg. Also, right MG showed high consistency with left MG and BFL.												
**Right**	MG	61.1(3.2)	67.6(3.3)	54.3(3.5)	47.9(4.4)	52.5(3.0)	60.5(4.0)	56.7(2.6)	55.4(2.7)	52.0(1.0)	50.0(1.3)	60.6(5.4)
		LG	68.6(5.2)	54.3(5.5)	52.7(4.2)	49.6(7.4)	56.1(3.6)	58.2(5.7)	56.6(8.2)	52.1(3.8)	51.8(4.5)	59.0(3.7)
			SOL	53.4(4.8)	49.2(1.4)	50.6(3.3)	56.6(3.3)	58.8(4.6)	57.3(6.8)	52.2(1.9)	49.1(1.5)	57.5(4.7)
				TA	51.6(5.5)	55.9(3.4)	50.5(1.9)	52.7(1.9)	54.8(2.4)	50.3(3.5)	47.1(4.4)	49.3(6.8)
					RF	57.2(9.5)	47.0(0.8)	47.5(2.7)	49.7(5.6)	51.4(4.5)	51.7(5.8)	51.6(6.6)
						BFL	51.6(3.6)	53.8(2.9)	58.5(2.0)	48.8(3.5)	48.0(6.1)	48.1(7.7)
**Left**							MG	64.7(2.7)	67.0(3.2)	49.0(4.5)	51.0(1.5)	58.2(6.4)
								LG	73.7(5.0)	48.3(2.4)	50.6(6.1)	54.8(4.6)
									SOL	46.3(4.4)	48.0(6.9)	54.4(7.7)
										TA	49.8(2.4)	54.0(3.4)
											RF	51.1(4.9)
												BFL
**On/off consistency of participant 8**. Consistency among triceps surae muscles and TA were high both in right and left leg. Right RF on/off was oppositely consistent with that of ipsilateral MG and LG, and with contralateral triceps surae muscles and TA, and also showed high consistency with contralateral RF and BFL. Left RF showed low consistency with left triceps surae muscles. In addition, left BFL on/off was consistent with that of right BFL and left RF.												
**Right**	MG	77.8(3.4)	74.8(6.4)	65.1(4.3)	38.8(8.1)	48.5(6.0)	52.2(7.7)	47.7(6.5)	49.4(3.4)	43.3(7.9)	41.3(8.2)	47.7(10.8)
		LG	77.2(5.9)	62.9(5.8)	39.5(9.0)	49.0(6.9)	50.8(6.1)	48.5(5.4)	49.9(4.3)	42.9(8.7)	42.0(7.6)	48.0(7.5)
			SOL	61.8(4.0)	41.6(7.7)	48.7(7.2)	49.0(4.6)	51.1(2.7)	49.0(5.4)	42.0(7.7)	40.7(5.2)	46.9(6.4)
				TA	46.4(8.5)	55.5(5.0)	47.9(1.6)	46.9(7.4)	49.7(4.0)	43.9(2.2)	47.0(7.4)	48.5(3.7)
					RF	58.8(8.8)	39.6(2.5)	34.1(9.1)	31.9(4.4)	39.5(5.5)	71.8(7.0)	63.6(11.4)
						BFL	49.2(5.5)	47.7(6.8)	46.3(6.1)	48.1(4.4)	57.3(7.6)	62.1(3.5)
**Left**							MG	67.4(6.7)	66.3(5.9)	67.5(5.3)	37.5(2.7)	49.3(7.1)
								LG	77.4(11.8)	72.8(6.2)	34.6(5.2)	43.0(8.6)
									SOL	71.9(7.6)	33.5(3.7)	40.5(8.1)
										TA	41.7(4.7)	47.6(8.2)
											RF	67.6(9.1)
												BFL
**Average value of on/off consistency for all participants**. Consistency among triceps surae muscles and TA were high both in right and left leg. Right BFL on/off was consistent with that of left MG, and left RF on/off was oppositely consistent with that of left LG and SOL.												
**Right**	MG	70.8(7.0)	70.8(7.0)	58.1(9.5)	45.7(9.1)	56.2(9.4)	59.4(8.4)	54.4(5.5)	53.6(6.6)	53.4(8.7)	46.1(6.8)	59.4(9.5)
		LG	74.5(6.9)	55.7(7.2)	43.8(10.0)	52.4(7.1)	53.7(4.9)	53.8(7.0)	54.4(8.1)	49.1(6.3)	46.3(7.0)	54.4(6.4)
			SOL	51.2(9.2)	41.5(9.9)	51.8(7.3)	53.6(5.7)	54.5(6.6)	54.6(7.8)	47.7(6.7)	43.4(6.4)	54.3(6.7)
				TA	55.3(9.4)	57.0(8.7)	54.6(8.2)	49.3(7.5)	49.6(7.6)	57.2(11.0)	52.3(8.8)	55.0(9.3)
					RF	54.5(8.1)	46.8(7.6)	44.1(8.6)	43.3(9.1)	52.1(9.9)	58.7(10.7)	53.4(7.5)
						BFL	61.5(10.1)	55.7(8.4)	55.9(8.5)	54.0(8.4)	48.8(8.2)	57.3(9.7)
**Left**							MG	68.2(5.3)	68.4(7.1)	58.4(8.9)	45.3(7.0)	59.2(10.4)
								LG	77.3(7.9)	56.1(10.3)	39.5(8.8)	51.2(9.6)
									SOL	52.6(11.3)	38.7(9.0)	49.8(9.5)
										TA	51.7(8.8)	55.2(7.0)
											RF	57.5(8.8)
												BFL

On/off consistency ratio (SD) [%] between different muscles were calculated for each participant. Consistency of 100 means that activation and inactivation of two muscles are absolutely consistent, and consistency of 0 means that activation and inactivation of two muscles are exactly opposite. We picked up pairs of muscles who showed relatively high (>60%) and low (<40%) on/off consistency.

The on/off distribution in the phase planes (Fig. 1) varied more widely compared with that in the torque planes (Fig. 2). A statistical test revealed that this variability was due to individual variation and laterality (Table 2). It might be assumed that this is due to the individual and lateral differences in the transfer lag of afferent feedback and the efferent control input via the musculoskeletal system. However, the variation of the time delay for sensory transduction, neural processing, transmission, and muscle activation during standing is around 20 ms^15^, which would not be sufficient to change on/off areas in the phase planes. In addition, it has been shown that there is a substantial amount of variability in the relationship between EMG activity and the force generated by a muscle, that is, electro-mechanical delay^42^. Therefore, it is possible that the on/off trigger timing of intermittent muscle activities (i.e., the reference value of the state point for each muscle) is modulated depending on individually or laterally different mechanical/structural properties of the body (such as segment length, joint viscoelasticity, or physiological cross-sectional area of muscles), so as to generate joint torque precisely in the direction of anatomical action (Fig. 2). Although the calculation precision of joint torques affect the reliability of Fig. 2, coordinates of on/off centers of most trials were close to 1 or more for some muscles (at least tricels surae muscles on the ankle and knee torque planes and thigh muscles on the knee torque plane). This means that on/off centers are close to the edge of on/off areas for such trials and indicates the less possibility that the variability of on/off centers is smaller than the precision of joint torques.

**Table 2:** Variability of on/off centers on phase and torque planes.

		Sub 1		Sub 2		Sub 3		Sub 4		Sub 5		Sub 6		Sub 7		Sub 8	
		θ	ω	θ	ω	θ	ω	θ	ω	θ	ω	θ	ω	θ	ω	θ	ω
**ON centers on the ANKLE phase plane**.																	
MG_L_	t	**3.87**	−1.07	0.75	1.72	2.58	**12.4**	**2.91**	**3.72**	**10.4**	**7.37**	**8.33**	1.07	**3.35**	2.44	**3.47**	2.24
	p	**<0.05**	0.34	0.50	0.16	0.061	**<0.05**	**<0.05**	**<0.05**	**<0.05**	**<0.05**	**<0.05**	0.35	**<0.05**	0.071	**<0.05**	0.089
LG_L_	t	2.02	−1.25	−0.12	**7.50**	**3.42**	0.58	1.79	**5.84**	**8.96**	**2.82**	2.42	**5.31**	−0.54	2.33	**2.82**	**5.10**
	p	0.11	0.28	0.91	**<0.05**	**<0.05**	0.60	0.15	**<0.05**	**<0.05**	**<0.05**	0.072	**<0.05**	0.62	0.080	**<0.05**	**<0.05**
SOL_L_	t	**2.81**	0.95	**2.90**	**2.93**	**5.28**	1.82	2.60	**3.79**	**6.29**	**2.80**	**4.89**	**3.64**	1.63	2.53	**2.84**	1.59
	p	**<0.05**	0.40	**<0.05**	**<0.05**	**<0.05**	0.14	0.060	**<0.05**	**<0.05**	**<0.05**	**<0.05**	**<0.05**	0.18	0.065	**<0.05**	0.19
TA_L_	t	−2.27	**−3.00**	−2.09	**−3.82**	−1.37	−1.94	**2.81**	−2.19	**2.98**	−0.83	−0.45	−1.33	0.62	0.63	0.92	−0.072
	p	0.086	**<0.05**	0.10	**<0.05**	0.24	0.12	**<0.05**	0.094	**<0.05**	0.45	0.67	0.26	0.57	0.56	0.41	0.95
RF_L_	t	−0.93	−1.53	**−6.09**	**−4.49**	**−3.34**	**−4.10**	−0.31	**−2.85**	−1.27	**4.55**	0.71	−1.88	0.82	−1.96	**−11.1**	**−4.22**
	p	0.41	0.20	**<0.05**	**<0.05**	**<0.05**	**<0.05**	0.77	**<0.05**	0.27	**<0.05**	0.52	0.13	0.46	0.12	**<0.05**	**<0.05**
BFL_L_	t	−0.82	0.086	−0.21	**−4.18**	−0.33	**−7.33**	0.82	−1.82	**3.53**	0.92	0.55	−1.38	1.35	−0.53	−2.24	**−3.18**
	p	0.46	0.94	0.85	**<0.05**	0.76	**<0.05**	0.46	0.14	**<0.05**	0.41	0.61	0.24	0.25	0.62	0.088	**<0.05**
MG_R_	t	**5.53**	**5.24**	1.38	**3.39**	**3.36**	**2.84**	**3.81**	−1.33	2.65	1.72	1.45	1.51	0.97	0.61	**3.64**	−1.78
	p	**<0.05**	**<0.05**	0.24	**<0.05**	**<0.05**	**<0.05**	**<0.05**	0.25	0.057	0.16	0.22	0.21	0.39	0.58	**<0.05**	0.15
LG_R_	t	**3.02**	**4.39**	2.27	**6.49**	**2.92**	1.13	**7.24**	0.12	1.66	−0.17	**4.97**	0.33	0.65	1.30	1.72	−2.21
	p	**<0.05**	**<0.05**	0.086	**<0.05**	**<0.05**	0.32	**<0.05**	0.91	0.17	0.87	**<0.05**	0.76	0.55	0.26	0.16	0.091
SOL_R_	t	**6.14**	**3.90**	2.71	**7.47**	2.20	**3.70**	**3.91**	0.61	2.03	0.24	**6.33**	2.24	1.04	1.18	1.63	**−3.51**
	p	**<0.05**	**<0.05**	0.054	**<0.05**	0.093	**<0.05**	**<0.05**	0.57	0.11	0.82	**<0.05**	0.088	0.36	0.30	0.18	**<0.05**
TA_R_	t	**−2.82**	**−4.96**	−1.04	−1.18	−0.69	−0.56	−0.35	−2.76	0.83	**−2.81**	−1.06	−1.36	−0.56	1.86	1.06	**−3.33**
	p	**<0.05**	**<0.05**	0.36	0.30	0.53	0.60	0.74	0.051	0.45	**<0.05**	0.35	0.25	0.60	0.14	0.35	**<0.05**
RF_R_	t	**−7.20**	**−12.1**	**−5.38**	**−6.78**	−1.94	−1.23	**−4.36**	−0.16	0.12	−0.97	0.10	0.36	2.04	1.13	**−8.00**	**−4.61**
	p	**<0.05**	**<0.05**	**<0.05**	**<0.05**	0.12	0.29	**<0.05**	0.88	0.91	0.39	0.92	0.73	0.11	0.32	**<0.05**	**<0.05**
BFL_R_	t	−0.59	**−2.76**	−0.14	**−5.88**	0.33	**−4.50**	2.76	−0.89	−1.05	0.99	2.49	−0.36	−0.80	2.74	**−6.03**	**−4.61**
	p	0.59	**<0.05**	0.90	**<0.05**	0.76	**<0.05**	0.051	0.42	0.35	0.38	0.067	0.73	0.47	0.052	**<0.05**	**<0.05**
**OFF centers on the ANKLE phase plane**.																	
MG_L_	t	**−3.70**	1.30	0.25	−1.35	−2.52	**−9.97**	**−6.27**	**−3.59**	**−6.01**	**−6.05**	**−7.91**	−1.86	−1.12	**−3.17**	**−3.43**	−1.76
	p	**<0.05**	0.26	0.82	0.25	0.065	**<0.05**	**<0.05**	**<0.05**	**<0.05**	**<0.05**	**<0.05**	0.14	0.33	**<0.05**	**<0.05**	0.15
LG_L_	t	−2.46	2.41	−0.012	**−5.95**	**−3.31**	−0.67	**−4.32**	**−7.75**	**−6.18**	−2.76	−2.51	**−4.99**	**2.78**	**−4.58**	**−2.81**	**−5.61**
	p	0.070	0.074	0.99	**<0.05**	**<0.05**	0.54	**<0.05**	**<0.05**	**<0.05**	0.051	0.066	**<0.05**	**<0.05**	**<0.05**	**<0.05**	**<0.05**
SOL_L_	t	**−3.80**	−0.47	**−3.22**	**−3.81**	**−4.16**	−1.95	−2.54	**−4.57**	**−3.75**	−2.51	**−4.50**	**−4.09**	−0.80	−2.40	**−2.98**	−1.56
	p	**<0.05**	0.66	**<0.05**	**<0.05**	**<0.05**	0.12	0.064	**<0.05**	**<0.05**	0.066	**<0.05**	**<0.05**	0.47	0.074	**<0.05**	0.19
TA_L_	t	1.35	**3.53**	1.62	**3.02**	1.29	2.40	−1.47	1.88	**−2.98**	0.81	0.038	1.37	1.71	−0.80	−1.26	−0.35
	p	0.25	**<0.05**	0.18	**<0.05**	0.27	0.074	0.22	0.13	**<0.05**	0.46	0.97	0.24	0.16	0.47	0.27	0.74
RF_L_	t	1.21	**2.85**	2.77	**4.55**	**3.88**	**5.03**	−0.027	2.38	1.07	**−5.55**	−0.26	1.10	−0.045	1.91	**6.75**	**5.11**
	p	0.29	**<0.05**	0.051	**<0.05**	**<0.05**	**<0.05**	0.98	0.076	0.34	**<0.05**	0.81	0.33	0.97	0.13	**<0.05**	**<0.05**
BFL_L_	t	0.92	0.54	−0.18	**3.06**	0.61	**8.52**	−1.85	1.66	**−3.44**	−1.55	−0.43	1.23	1.27	−1.64	2.43	**4.08**
	p	0.41	0.62	0.87	**<0.05**	0.57	**<0.05**	0.14	0.17	**<0.05**	0.20	0.69	0.29	0.27	0.18	0.072	**<0.05**
MG_R_	t	**−4.44**	**−4.15**	−0.66	**−4.42**	−2.73	−2.47	**−3.12**	1.07	−2.60	−1.75	−2.06	−2.11	0.59	−0.46	−1.88	2.36
	p	**<0.05**	**<0.05**	0.54	**<0.05**	0.053	0.069	**<0.05**	0.35	0.060	0.16	0.11	0.10	0.58	0.67	0.13	0.078
LG_R_	t	**−2.85**	−2.49	**−3.06**	**−5.75**	−2.56	−1.16	**−15.2**	0.19	−1.71	0.81	**−3.10**	−0.61	−0.36	−1.39	−1.82	2.08
	p	**<0.05**	0.068	**<0.05**	**<0.05**	0.063	0.31	**<0.05**	0.86	0.16	0.47	**<0.05**	0.57	0.74	0.24	0.14	0.11
SOL_R_	t	**−5.44**	**−3.65**	**−2.82**	**−10.7**	−1.38	**−4.71**	**−7.27**	−0.66	−1.71	0.25	**−5.18**	−2.27	0.59	−1.70	−1.50	**3.30**
	p	**<0.05**	**<0.05**	**<0.05**	**<0.05**	0.24	**<0.05**	**<0.05**	0.55	0.16	0.82	**<0.05**	0.085	0.59	0.16	0.21	**<0.05**
TA_R_	t	2.28	**3.39**	1.07	1.31	1.05	0.36	−0.90	1.77	−1.45	2.26	1.02	1.55	1.98	**−2.89**	−1.02	**3.43**
	p	0.084	**<0.05**	0.34	0.26	0.35	0.73	0.42	0.15	0.22	0.087	0.36	0.20	0.12	**<0.05**	0.36	**<0.05**
RF_R_	t	**6.56**	**8.53**	1.98	**6.39**	2.07	1.35	**3.62**	0.19	−0.66	0.60	0.76	−0.67	0.080	−1.95	**5.88**	**4.61**
	p	**<0.05**	**<0.05**	0.12	**<0.05**	0.11	0.25	**<0.05**	0.86	0.55	0.58	0.49	0.54	0.94	0.12	**<0.05**	**<0.05**
BFL_R_	t	1.15	2.29	0.22	**4.36**	−0.46	**6.17**	**−2.99**	0.15	1.04	−0.31	−2.04	0.39	0.85	**−3.36**	**5.84**	**5.15**
	p	0.32	0.084	0.84	**<0.05**	0.67	**<0.05**	**<0.05**	0.88	0.36	0.77	0.11	0.72	0.44	**<0.05**	**<0.05**	**<0.05**
**ON centers on the KNEE phase plane**.																	
MG_L_	t	2.67	**2.51**	**3.34**	1.84	1.98	**3.70**	2.01	**3.22**	1.87	1.62	2.24	1.54	**4.20**	**6.19**	0.80	1.49
	p	0.056	**<0.05**	**<0.05**	0.14	0.12	**<0.05**	0.11	**<0.05**	0.14	0.18	0.089	0.20	**<0.05**	**<0.05**	0.47	0.21
LG_L_	t	2.75	**2.79**	**3.32**	2.38	**2.94**	**4.80**	1.47	**4.00**	0.69	2.62	−0.26	**3.27**	2.40	**5.16**	0.20	2.35
	p	0.051	**<0.05**	**<0.05**	0.076	**<0.05**	**<0.05**	0.21	**<0.05**	0.53	0.059	0.81	**<0.05**	0.075	**<0.05**	0.85	0.078
SOL_L_	t	**2.79**	**3.26**	2.52	**7.02**	0.56	**4.35**	1.61	**3.24**	0.21	2.12	−0.89	1.93	2.39	**6.71**	0.22	1.36
	p	**<0.05**	**<0.05**	0.065	**<0.05**	0.61	**<0.05**	0.18	**<0.05**	0.85	0.10	0.43	0.13	0.075	**<0.05**	0.83	0.24
TA_L_	t	0.36	−1.57	−0.98	−2.72	1.51	1.11	1.57	**3.61**	0.42	2.01	0.91	0.35	0.42	**4.05**	1.62	1.31
	p	0.73	0.19	0.38	0.053	0.20	0.33	0.19	**<0.05**	0.69	0.11	0.41	0.75	0.69	**<0.05**	0.18	0.26
RF_L_	t	−0.11	**−3.96**	−2.04	**−7.92**	−2.70	**−3.80**	−0.20	−0.63	**−2.95**	−0.32	−1.78	1.47	−1.64	**−3.83**	−1.79	−1.75
	p	0.92	**<0.05**	0.11	**<0.05**	0.054	**<0.05**	0.85	0.56	**<0.05**	0.77	0.15	0.22	0.18	**<0.05**	0.15	0.16
BFL_L_	t	0.18	−1.40	**5.24**	**4.71**	−0.53	1.54	1.12	**7.42**	**4.76**	**5.45**	1.53	**3.21**	−1.03	1.17	**2.78**	1.92
	p	0.87	0.23	**<0.05**	**<0.05**	0.63	0.20	0.33	**<0.05**	**<0.05**	**<0.05**	0.20	**<0.05**	0.36	0.31	**<0.05**	0.13
MG_R_	t	1.85	**2.88**	**2.78**	**7.85**	0.47	**3.51**	2.42	**4.40**	**2.90**	**9.71**	1.80	2.69	**4.84**	**9.06**	1.50	**6.32**
	p	0.14	**<0.05**	**<0.05**	**<0.05**	0.66	**<0.05**	0.073	**<0.05**	**<0.05**	**<0.05**	0.15	0.055	**<0.05**	**<0.05**	0.21	**<0.05**
LG_R_	t	**4.59**	**6.55**	0.71	**12.2**	**3.89**	**4.96**	**5.41**	**7.38**	0.90	**8.23**	1.47	**3.55**	**2.86**	**11.6**	1.54	**5.91**
	p	**<0.05**	**<0.05**	0.52	**<0.05**	**<0.05**	**<0.05**	**<0.05**	**<0.05**	0.42	**<0.05**	0.21	**<0.05**	**<0.05**	**<0.05**	0.20	**<0.05**
SOL_R_	t	**7.05**	**4.29**	1.49	**9.18**	**4.87**	2.77	**5.33**	**6.87**	0.35	**13.5**	−0.21	2.26	2.13	**9.92**	2.60	**10.0**
	p	**<0.05**	**<0.05**	0.21	**<0.05**	**<0.05**	0.050	**<0.05**	**<0.05**	0.74	**<0.05**	0.85	0.086	0.10	**<0.05**	0.060	**<0.05**
TA_R_	t	−1.60	−0.60	−0.66	1.23	2.21	**4.18**	0.88	0.29	1.81	0.82	1.16	0.34	2.40	**−5.25**	2.07	**7.75**
	p	0.18	0.58	0.54	0.29	0.091	**<0.05**	0.43	0.78	0.14	0.46	0.31	0.75	0.074	**<0.05**	0.11	**<0.05**
RF_R_	t	0.098	**−3.11**	0.14	**−7.71**	−1.13	**−3.05**	**−5.47**	**−8.35**	−1.08	−2.30	−1.17	−1.12	**−3.13**	**−3.47**	−0.21	−2.11
	p	0.93	**<0.05**	0.90	**<0.05**	0.32	**<0.05**	**<0.05**	**<0.05**	0.34	0.083	0.31	0.33	**<0.05**	**<0.05**	0.84	0.10
BFL_R_	t	**3.72**	**6.06**	2.59	**3.65**	−0.70	2.41	2.31	1.72	−1.51	**−4.22**	2.28	**2.99**	1.85	1.89	0.72	1.90
	p	**<0.05**	**<0.05**	0.061	**<0.05**	0.52	0.074	0.082	0.16	0.21	**<0.05**	0.085	**<0.05**	0.14	0.13	0.51	0.13
**OFF centers on the KNEE phase plane**.																	
MG_L_	t	**−3.10**	**−3.10**	**−3.84**	−1.13	−2.24	**−5.53**	−1.82	**−2.81**	**−6.03**	−0.84	−2.57	−2.04	**−3.98**	**−10.7**	−1.73	−1.41
	p	**<0.05**	**<0.05**	**<0.05**	0.32	0.089	**<0.05**	0.14	**<0.05**	**<0.05**	0.45	0.062	0.11	**<0.05**	**<0.05**	0.16	0.23
LG_L_	t	**−4.28**	−2.48	**−4.15**	−2.26	**−2.97**	**−5.54**	−2.39	**−3.40**	−2.05	−2.41	1.16	**−2.78**	**−3.00**	**−5.27**	−2.32	−1.83
	p	**<0.05**	0.069	**<0.05**	0.087	**<0.05**	**<0.05**	0.075	**<0.05**	0.11	0.073	0.31	**<0.05**	**<0.05**	**<0.05**	0.081	0.14
SOL_L_	t	**−2.95**	**−3.13**	−2.51	**−3.03**	−0.85	**−4.16**	−1.47	**−2.83**	−0.95	−1.80	0.68	1.69	−2.71	**−6.67**	−0.63	−1.11
	p	**<0.05**	**<0.05**	0.066	**<0.05**	0.44	**<0.05**	0.22	**<0.05**	0.40	0.15	0.53	0.17	0.053	**<0.05**	0.56	0.33
TA_L_	t	−0.82	2.08	0.39	**3.12**	−1.72	−0.79	−2.45	**−3.19**	−0.41	−2.17	−0.93	−0.29	**−2.94**	**−3.46**	−2.08	−1.00
	p	0.46	0.11	0.72	**<0.05**	0.16	0.47	0.071	**<0.05**	0.70	0.095	0.41	0.78	**<0.05**	**<0.05**	0.11	0.37
RF_L_	t	−2.04	**3.23**	0.99	**7.34**	0.77	**4.56**	−0.033	0.60	2.28	0.22	1.49	−0.90	0.80	**4.47**	1.60	1.81
	p	0.11	**<0.05**	0.38	**<0.05**	0.48	**<0.05**	0.98	0.58	0.085	0.84	0.21	0.42	0.47	**<0.05**	0.19	0.14
BFL_L_	t	−1.66	1.12	**−8.26**	**−7.11**	0.26	−1.18	−1.91	**−3.39**	**−7.99**	**−10.8**	−1.92	**−4.34**	−1.46	−0.047	**−2.92**	−2.15
	p	0.17	0.32	**<0.05**	**<0.05**	0.81	0.30	0.13	**<0.05**	**<0.05**	**<0.05**	0.13	**<0.05**	0.22	0.97	**<0.05**	0.098
MG_R_	t	−1.85	−2.75	**−3.95**	**−12.4**	−0.44	**−4.70**	**−3.31**	**−7.78**	**−3.20**	**−13.3**	−1.87	**−4.18**	**−4.60**	**−7.31**	−1.83	**−7.93**
	p	0.14	0.051	**<0.05**	**<0.05**	0.69	**<0.05**	**<0.05**	**<0.05**	**<0.05**	**<0.05**	0.14	**<0.05**	**<0.05**	**<0.05**	0.14	**<0.05**
LG_R_	t	**−3.03**	**−6.34**	−1.77	**−10.3**	−2.29	**−5.19**	**−7.77**	**−10.5**	−1.90	**−4.96**	−1.54	−2.76	**−2.88**	**−9.69**	−1.52	**−5.75**
	p	**<0.05**	**<0.05**	0.15	**<0.05**	0.084	**<0.05**	**<0.05**	**<0.05**	0.13	**<0.05**	0.20	0.051	**<0.05**	**<0.05**	0.20	**<0.05**
SOL_R_	t	**−5.04**	**−3.80**	−1.62	**−8.64**	**−2.98**	**−2.78**	**−6.94**	**−6.35**	**−3.08**	**−7.39**	0.20	−2.42	−2.68	**−8.32**	**−2.87**	**−11.9**
	p	**<0.05**	**<0.05**	0.18	**<0.05**	**<0.05**	**<0.05**	**<0.05**	**<0.05**	**<0.05**	**<0.05**	0.85	0.073	0.055	**<0.05**	**<0.05**	**<0.05**
TA_R_	t	0.13	0.69	0.20	−1.34	**−8.58**	**−3.03**	−1.55	−0.97	−1.68	−1.01	−1.49	−0.61	**−3.96**	**5.30**	−2.27	**−6.43**
	p	0.91	0.53	0.85	0.25	**<0.05**	**<0.05**	0.20	0.39	0.17	0.37	0.21	0.57	**<0.05**	**<0.05**	0.086	**<0.05**
RF_R_	t	−0.68	3.37	−0.76	8.41	1.01	3.90	5.11	8.15	0.49	5.10	0.61	1.40	0.47	3.95	0.017	1.87
	p	0.53	<0.05	0.49	<0.05	0.37	<0.05	<0.05	<0.05	0.65	<0.05	0.57	0.23	0.66	<0.05	0.99	0.13
BFL_R_	t	−3.53	−5.20	−5.10	−3.60	1.01	−2.14	−2.93	−1.94	0.30	3.34	−2.57	−3.43	−2.73	−1.48	−0.70	−1.91
	p	<0.05	<0.05	<0.05	<0.05	0.37	0.099	<0.05	0.12	0.78	<0.05	0.062	<0.05	0.052	0.21	0.52	0.13
**ON centers on the HIP phase plane**.																	
MG_L_	t	**−2.48**	−1.67	1.22	**−3.08**	−1.88	−2.70	−1.24	**−3.76**	−1.13	−1.21	−1.61	−1.25	−0.24	0.96	−0.19	2.03
	p	**<0.05**	0.17	0.29	**<0.05**	0.13	0.054	0.28	**<0.05**	0.32	0.29	0.18	0.28	0.82	0.39	0.86	0.11
LG_L_	t	0.093	−2.23	0.0082	**−3.59**	**−3.38**	**−4.49**	−0.34	**−3.95**	−1.64	−2.52	−1.60	**−3.00**	2.00	0.86	0.93	0.65
	p	0.93	0.089	0.99	**<0.05**	**<0.05**	**<0.05**	0.75	**<0.05**	0.18	0.066	0.18	**<0.05**	0.12	0.44	0.41	0.55
SOL_L_	t	−0.75	−1.94	0.37	**−3.76**	−2.27	**−3.07**	−1.04	**−4.71**	−0.72	−2.71	**−3.06**	**−3.32**	0.34	−1.77	0.84	2.61
	p	0.50	0.12	0.73	**<0.05**	0.086	**<0.05**	0.36	**<0.05**	0.51	0.054	**<0.05**	**<0.05**	0.75	0.15	0.45	0.059
TA_L_	t	0.58	0.23	−1.13	2.31	0.95	1.84	−0.40	0.62	−0.86	0.50	1.67	**2.91**	−0.49	0.29	−0.68	2.40
	p	0.59	0.83	0.32	0.082	0.40	0.14	0.71	0.57	0.44	0.64	0.17	**<0.05**	0.65	0.79	0.54	0.074
RF_L_	t	−1.41	−0.32	0.26	0.015	**3.39**	**2.94**	0.39	1.58	2.75	0.032	**3.02**	1.48	−0.98	2.02	−0.37	−1.13
	p	0.23	0.77	0.81	0.99	**<0.05**	**<0.05**	0.71	0.19	0.051	0.98	**<0.05**	0.21	0.38	0.11	0.73	0.32
BFL_L_	t	−1.15	**−3.72**	1.53	1.28	−0.0053	2.44	0.97	−1.00	−1.71	−0.53	−2.26	**−3.93**	−1.03	1.04	−0.55	1.01
	p	0.31	**<0.05**	0.20	0.27	0.99	0.071	0.39	0.38	0.16	0.63	0.087	**<0.05**	0.36	0.36	0.61	0.27
MG_R_	t	0.21	**−3.37**	0.27	−2.66	−1.68	**−4.38**	−0.43	−1.83	−1.92	−1.94	−0.89	**−5.51**	2.23	2.20	−0.91	−0.81
	p	0.84	**<0.05**	0.80	0.057	0.17	**<0.05**	0.69	0.14	0.13	0.12	0.43	**<0.05**	0.090	0.093	0.41	0.47
LG_R_	t	−0.27	**−3.47**	0.19	**−2.88**	**−3.01**	**−4.83**	**−6.12**	−2.36	0.44	−2.57	**−3.44**	**−5.46**	0.47	0.60	−0.011	−1.49
	p	0.80	**<0.05**	0.86	**<0.05**	**<0.05**	**<0.05**	**<0.05**	0.077	0.68	0.062	**<0.05**	**<0.05**	0.66	0.58	0.99	0.21
SOL_R_	t	−0.031	**−3.22**	−0.31	**−3.63**	**−4.32**	**−5.22**	−1.20	−2.51	1.20	**−3.50**	−2.11	**−8.31**	−0.42	−0.047	−0.40	−2.50
	p	0.98	**<0.05**	0.78	**<0.05**	**<0.05**	**<0.05**	0.30	0.066	0.29	**<0.05**	0.10	**<0.05**	0.70	0.96	0.71	0.067
TA_R_	t	0.11	2.67	−0.27	1.89	−1.60	**−5.64**	−0.12	**3.78**	0.24	1.50	0.077	0.58	1.08	0.49	−1.55	**−5.10**
	p	0.92	0.056	0.80	0.30	0.18	**<0.05**	0.91	**<0.05**	0.82	0.21	0.94	0.59	0.34	0.65	0.20	**<0.05**
RF_R_	t	−1.39	1.21	0.088	0.33	0.96	2.49	1.52	1.31	−1.73	0.19	2.27	1.94	−1.90	0.025	−1.42	−1.04
	p	0.24	0.29	0.93	0.76	0.39	0.067	0.20	0.26	0.16	0.86	0.085	0.12	0.13	0.98	0.22	0.36
BFL_R_	t	0.60	1.55	−0.12	0.54	0.16	−0.25	−1.62	−1.96	0.081	−1.91	0.81	−0.50	1.09	0.26	−0.32	−0.82
	p	0.58	0.20	0.91	0.62	0.88	0.82	0.18	0.12	0.94	0.13	0.46	0.64	0.34	0.81	0.77	0.46
**OFF centers on the HIP phase plane**.																	
MG_L_	t	2.41	1.26	−0.025	2.48	1.68	**3.16**	**4.67**	**2.87**	1.52	0.30	**3.70**	0.83	−0.56	−0.57	0.49	−1.91
	p	0.074	0.28	0.98	0.068	0.17	**<0.05**	**<0.05**	**<0.05**	0.20	0.78	**<0.05**	0.45	0.60	0.60	0.65	0.13
LG_L_	t	1.75	1.91	0.067	**3.35**	**3.23**	**4.96**	**3.59**	**3.22**	**2.81**	2.07	2.74	1.99	**−3.77**	0.49	1.77	−0.81
	p	0.16	0.13	0.95	**<0.05**	**<0.05**	**<0.05**	**<0.05**	**<0.05**	**<0.05**	0.11	0.052	0.12	**<0.05**	0.65	0.15	0.47
SOL_L_	t	**3.75**	1.70	0.23	**3.53**	2.09	**2.96**	1.30	**3.50**	1.82	2.34	**3.96**	**3.04**	−1.38	1.74	−0.28	−2.63
	p	**<0.05**	0.16	0.83	**<0.05**	0.11	**<0.05**	0.26	**<0.05**	0.14	0.079	**<0.05**	**<0.05**	0.24	0.16	0.79	0.058
TA_L_	t	0.074	−0.76	0.71	−2.60	−0.66	−1.54	1.77	−0.87	0.83	−0.64	−1.84	**−3.28**	−1.76	0.42	0.83	−2.36
	p	0.94	0.49	0.52	0.060	0.55	0.20	0.15	0.43	0.45	0.56	0.14	**<0.05**	0.15	0.69	0.45	0.078
RF_L_	t	1.91	0.68	1.08	−0.078	**−2.89**	**−4.52**	0.30	−1.30	−2.17	−0.23	−1.62	−1.92	0.17	−1.45	0.81	0.92
	p	0.13	0.54	0.34	0.94	**<0.05**	**<0.05**	0.78	0.26	0.096	0.83	0.18	0.13	0.87	0.22	0.46	0.41
BFL_L_	t	0.98	**3.03**	−0.46	−1.58	−0.15	−2.47	−0.53	0.36	1.97	0.33	**3.79**	**4.01**	−1.30	−0.12	0.75	−0.93
	p	0.38	**<0.05**	0.67	0.19	0.89	0.069	0.62	0.74	0.12	0.76	**<0.05**	**<0.05**	0.26	0.91	0.49	0.41
MG_R_	t	0.25	**2.92**	−0.61	**2.85**	1.80	**5.56**	1.60	1.99	2.08	2.27	0.66	**16.8**	**−4.41**	−1.95	1.39	1.46
	p	0.81	**<0.05**	0.57	**<0.05**	0.15	**<0.05**	0.18	0.11	0.11	0.086	0.55	**<0.05**	**<0.05**	0.12	0.24	0.22
LG_R_	t	−0.026	**3.62**	−0.16	2.66	**2.86**	**5.11**	**7.82**	**2.82**	2.14	2.32	2.66	**3.79**	−1.05	−0.81	0.26	1.40
	p	0.98	**<0.05**	0.88	0.056	**<0.05**	**<0.05**	**<0.05**	**<0.05**	0.099	0.081	0.056	**<0.05**	0.35	0.46	0.81	0.23
SOL_R_	t	0.074	**3.38**	0.41	**3.64**	1.83	**7.07**	**2.80**	**3.07**	**7.80**	**4.49**	2.19	**6.00**	−1.58	0.79	1.63	2.34
	p	0.94	**<0.05**	0.70	**<0.05**	0.14	**<0.05**	**<0.05**	**<0.05**	**<0.05**	**<0.05**	0.094	**<0.05**	0.19	0.47	0.18	0.080
TA_R_	t	0.86	−2.36	0.25	−1.56	2.30	**3.64**	1.38	−2.39	0.46	−1.21	0.40	−0.44	−2.24	0.082	1.91	**4.18**
	p	0.44	0.078	0.82	0.19	0.083	**<0.05**	0.24	0.075	0.67	0.29	0.71	0.68	0.088	0.94	0.13	**<0.05**
RF_R_	t	1.40	−1.30	0.89	−0.32	−1.08	−2.50	−0.63	−1.02	1.73	−0.29	−2.12	−1.96	−0.27	0.55	1.84	1.23
	p	0.24	0.26	0.42	0.76	0.34	0.067	0.57	0.36	0.16	0.78	0.10	0.12	0.80	0.61	0.14	0.29
BFL_R_	t	−0.45	−1.30	0.56	−0.52	0.76	−0.76	1.65	1.62	0.97	0.99	0.45	−0.11	−1.33	−0.54	0.22	0.92
	p	0.67	0.26	0.61	0.63	0.49	0.49	0.17	0.18	0.39	0.38	0.68	0.92	0.25	0.62	0.84	0.41
**ON centers on the ANKLE torque plane**.																	
		**T**	**dT/dt**	**T**	**dT/dt**	**T**	**dT/dt**	**T**	**dT/dt**	**T**	**dT/dt**	**T**	**dT/dt**	**T**	**dT/dt**	**T**	**dT/dt**
MG_L_	t	−12.0	−6.41	−1.38	−1.54	−5.11	−6.01	−2.92	−5.99	−6.33	−14.4	−11.2	−6.44	−12.0	−13.1	−3.30	−4.17
	p	<0.05	<0.05	0.24	0.20	<0.05	<0.05	<0.05	<0.05	<0.05	<0.05	<0.05	<0.05	<0.05	<0.05	<0.05	<0.05
LG_L_	t	−3.73	−2.79	−0.76	−4.04	−4.31	−9.84	−2.07	−6.86	−2.40	−8.70	−3.66	−10.5	−1.51	−10.4	−3.03	−5.26
	p	<0.05	<0.05	0.49	<0.05	<0.05	<0.05	0.11	<0.05	0.074	<0.05	<0.05	<0.05	0.20	<0.05	<0.05	<0.05
SOL_L_	t	−4.56	−14.0	−4.02	−6.58	−3.81	−9.30	−2.25	−6.78	−1.36	−8.58	−6.79	−8.79	−6.91	−9.64	−3.49	−5.21
	p	<0.05	<0.05	<0.05	<0.05	<0.05	<0.05	0.087	<0.05	0.25	<0.05	<0.05	<0.05	<0.05	<0.05	<0.05	<0.05
TA_L_	t	1.61	5.03	2.20	3.45	0.053	−0.13	−2.25	−1.15	−1.98	−2.45	−0.18	0.38	−1.30	−3.30	−4.39	−8.01
	p	0.18	<0.05	0.092	<0.05	0.96	0.90	0.087	0.32	0.12	0.071	0.86	0.72	0.26	<0.05	<0.05	<0.05
RF_L_	t	1.19	2.42	5.87	6.15	6.42	12.0	0.28	1.12	2.47	−0.44	1.65	1.64	−0.16	8.61	4.80	9.36
	p	0.30	0.072	<0.05	<0.05	<0.05	<0.05	0.80	0.33	0.069	0.68	0.17	0.18	0.88	<0.05	<0.05	<0.05
BFL_L_	t	0.87	1.53	−2.66	−1.33	0.59	0.28	−1.76	−7.76	−14.6	−14.4	−4.19	−5.48	−2.27	−0.91	0.31	8.39
	p	0.44	0.20	0.057	0.26	0.59	0.80	0.15	<0.05	<0.05	<0.05	<0.05	<0.05	0.086	0.42	0.77	<0.05
MG_R_	t	−8.14	−7.49	−2.60	−8.21	−18.2	−5.83	−5.23	−5.87	−6.31	−9.06	−7.42	−6.26	−7.08	−9.88	−2.33	−9.48
	p	<0.05	<0.05	0.060	<0.05	<0.05	<0.05	<0.05	<0.05	<0.05	<0.05	<0.05	<0.05	<0.05	<0.05	0.080	<0.05
LG_R_	t	−4.77	−10.4	−3.69	−13.5	−6.85	−7.41	−6.46	−9.97	−2.46	−12.9	−6.65	−4.97	−2.27	−21.0	−2.52	−9.09
	p	<0.05	<0.05	<0.05	<0.05	<0.05	<0.05	<0.05	<0.05	0.070	<0.05	<0.05	<0.05	0.086	<0.05	0.065	<0.05
SOL_R_	t	−9.43	−12.5	−4.40	−11.3	−11.1	−5.21	−5.54	−7.18	−2.03	−27.2	−13.8	−6.14	−2.85	−11.1	−4.04	−10.2
	p	<0.05	<0.05	<0.05	<0.05	<0.05	<0.05	<0.05	<0.05	0.11	<0.05	<0.05	<0.05	<0.05	<0.05	<0.05	<0.05
TA_R_	t	3.39	6.19	1.35	1.30	−2.92	−10.8	−0.23	0.48	−3.98	−1.41	0.15	0.77	−0.88	2.17	−2.82	−9.67
	p	<0.05	<0.05	0.25	0.26	<0.05	<0.05	0.83	0.66	<0.05	0.23	0.89	0.48	0.43	0.096	<0.05	<0.05
RF_R_	t	5.32	19.9	4.74	9.28	4.53	3.06	6.82	6.19	1.19	2.47	1.33	1.17	−1.56	3.19	5.12	9.87
	p	<0.05	<0.05	<0.05	<0.05	<0.05	<0.05	<0.05	<0.05	0.30	0.069	0.25	0.31	0.19	<0.05	<0.05	<0.05
BFL_R_	t	−1.79	−2.50	−1.59	1.18	0.50	−1.87	−2.70	−1.02	2.28	1.62	−6.34	−2.07	−0.29	−2.67	1.39	3.00
	p	0.15	0.067	0.19	0.30	0.64	0.13	0.054	0.37	0.085	0.18	<0.05	0.11	0.79	0.056	0.24	<0.05
**OFF centers on the ANKLE torque plane**.																	
MG_L_	t	11.7	9.16	0.34	0.75	8.25	14.1	2.80	5.04	7.94	5.50	9.89	8.43	4.58	14.6	4.10	5.46
	p	<0.05	<0.05	0.75	0.49	<0.05	<0.05	<0.05	<0.05	<0.05	<0.05	<0.05	<0.05	<0.05	<0.05	<0.05	<0.05
LG_L_	t	5.17	2.53	0.87	3.42	4.31	10.8	3.26	5.43	3.15	37.7	3.08	7.62	0.12	10.2	3.53	4.45
	p	<0.05	0.065	0.43	<0.05	<0.05	<0.05	<0.05	<0.05	<0.05	<0.05	<0.05	<0.05	0.91	<0.05	<0.05	<0.05
SOL_L_	t	6.47	25.8	3.34	4.13	3.78	8.38	1.93	5.57	1.69	17.4	6.76	7.70	6.70	11.6	3.19	5.33
	p	<0.05	<0.05	<0.05	<0.05	<0.05	<0.05	0.13	<0.05	0.17	<0.05	<0.05	<0.05	<0.05	<0.05	<0.05	<0.05
TA_L_	t	−0.91	−3.89	−2.02	−2.79	0.66	0.20	2.29	1.69	1.97	2.55	0.47	−0.32	−1.19	3.34	4.86	5.35
	p	0.41	<0.05	0.11	<0.05	0.55	0.85	0.084	0.17	0.12	<0.05	0.66	0.77	0.30	<0.05	<0.05	<0.05
RF_L_	t	−0.86	−2.64	−4.49	−6.92	−5.58	−8.61	−0.082	−1.08	−2.30	0.70	−1.12	−1.26	−0.42	−6.58	−3.77	−5.99
	p	0.44	0.058	<0.05	<0.05	<0.05	<0.05	0.94	0.34	0.083	0.62	0.33	0.28	0.70	<0.05	<0.05	<0.05
BFL_L_	t	−0.59	−1.66	3.18	0.75	−0.78	−0.70	2.70	3.19	11.1	6.44	5.55	5.29	−0.90	0.83	−0.67	−5.37
	p	0.59	0.17	<0.05	0.49	0.48	0.52	0.054	<0.05	<0.05	<0.05	<0.05	<0.05	0.42	0.46	0.54	<0.05
MG_R_	t	4.85	9.54	2.62	9.32	6.86	10.7	8.48	8.67	7.28	37.7	6.12	14.6	4.38	7.94	2.27	11.2
	p	<0.05	<0.05	0.059	<0.05	<0.05	<0.05	<0.05	<0.05	<0.05	<0.05	<0.05	<0.05	<0.05	<0.05	0.086	<0.05
LG_R_	t	5.09	5.55	4.42	9.35	5.30	6.89	11.5	18.5	3.18	5.91	3.75	3.56	2.38	10.5	2.49	11.5
	p	<0.05	<0.05	<0.05	<0.05	<0.05	<0.05	<0.05	<0.05	<0.05	<0.05	<0.05	<0.05	0.076	<0.05	0.067	<0.05
SOL_R_	t	7.07	8.14	3.97	9.02	8.63	5.62	8.78	10.8	3.74	9.70	7.22	5.01	4.57	7.43	3.79	10.7
	p	<0.05	<0.05	<0.05	<0.05	<0.05	<0.05	<0.05	<0.05	<0.05	<0.05	<0.05	<0.05	<0.05	<0.05	<0.05	<0.05
TA_R_	t	−3.08	−5.39	−1.23	−1.35	3.26	5.41	1.16	0.38	3.64	1.66	0.15	−0.55	−0.24	−1.88	2.67	7.93
	p	<0.05	<0.05	0.29	0.25	<0.05	<0.05	0.31	0.72	<0.05	0.11	0.89	0.61	0.82	0.13	0.056	<0.05
RF_R_	t	−6.18	−18.6	−2.80	−9.51	−4.51	−2.86	−5.76	−5.34	−0.65	−4.30	−1.18	−0.76	−0.19	−3.69	−3.90	−6.11
	p	<0.05	<0.05	<0.05	<0.05	<0.05	<0.05	<0.05	<0.05	0.55	<0.05	0.30	0.49	0.86	<0.05	<0.05	<0.05
BFL_R_	t	1.91	2.95	0.73	−1.31	−0.70	1.39	2.91	1.33	−1.23	−1.62	3.41	3.71	0.64	2.50	−1.61	−3.12
	p	0.13	<0.05	0.50	0.26	0.52	0.24	<0.05	0.26	0.29	0.17	<0.05	<0.05	0.56	0.067	0.18	<0.05
**ON centers on the KNEE torque plane**.																	
MG_L_	t	−11.9	−5.96	−1.43	−1.52	−5.45	−5.95	−2.87	−5.82	−5.87	−14.1	−10.8	−6.21	−14.5	−12.5	−3.15	−3.97
	p	<0.05	<0.05	0.23	0.20	<0.05	<0.05	<0.05	<0.05	<0.05	<0.05	<0.05	<0.05	<0.05	<0.05	<0.05	<0.05
LG_L_	t	−3.89	−2.70	−0.84	−3.83	−4.39	−9.39	−2.08	−6.57	−2.18	−8.44	−3.92	−10.9	−2.16	−9.40	−2.98	−4.97
	p	<0.05	0.054	0.45	<0.05	<0.05	<0.05	0.11	<0.05	0.095	<0.05	<0.05	<0.05	0.097	<0.05	<0.05	<0.05
SOL_L_	t	−4.83	−13.0	−4.04	−6.83	−3.58	−8.93	−2.23	−6.53	−1.21	−7.63	−6.84	−8.54	−8.15	−10.6	−3.34	−4.99
	p	<0.05	<0.05	<0.05	<0.05	<0.05	<0.05	0.090	<0.05	0.29	<0.05	<0.05	<0.05	<0.05	<0.05	<0.05	<0.05
TA_L_	t	1.52	4.89	2.20	3.39	−0.48	−0.33	−2.20	−1.33	−1.82	−2.51	−0.26	0.085	−1.55	−3.35	−4.51	−8.46
	p	0.20	<0.05	0.093	<0.05	0.66	0.76	0.092	0.26	0.14	0.066	0.81	0.94	0.20	<0.05	<0.05	<0.05
RF_L_	t	1.21	2.51	5.75	6.66	6.90	10.7	0.27	1.03	2.53	−0.37	1.66	1.37	0.11	6.37	4.52	9.86
	p	0.29	0.066	<0.05	<0.05	<0.05	<0.05	0.80	0.36	0.065	0.73	0.17	0.24	0.92	<0.05	<0.05	<0.05
BFL_L_	t	0.86	1.40	−2.84	−1.44	0.62	0.046	−1.78	−7.83	−12.1	−17.6	−3.94	−5.48	−2.44	−0.77	0.17	6.42
	p	0.44	0.23	<0.05	0.22	0.57	0.97	0.15	<0.05	<0.05	<0.05	<0.05	<0.05	0.071	0.48	0.87	<0.05
MG_R_	t	−8.15	−6.34	−2.71	−8.25	−23.2	−5.67	−4.91	−5.86	−6.75	−9.74	−7.32	−6.02	−7.52	−9.91	−2.27	−9.28
	p	<0.05	<0.05	0.054	<0.05	<0.05	<0.05	<0.05	<0.05	<0.05	<0.05	<0.05	<0.05	<0.05	<0.05	0.086	<0.05
LG_R_	t	−4.97	−9.47	−3.83	−13.4	−7.03	−7.52	−6.38	−10.9	−2.31	−13.7	−6.73	−5.23	−2.60	−23.0	−2.47	−8.29
	p	<0.05	<0.05	<0.05	<0.05	<0.05	<0.05	<0.05	<0.05	0.082	<0.05	<0.05	<0.05	0.060	<0.05	0.069	<0.05
SOL_R_	t	−9.84	−14.4	−4.52	−11.2	−12.7	−5.18	−5.65	−7.70	−1.86	−37.8	−14.5	−6.55	−3.43	−10.4	−4.00	−10.6
	p	<0.05	<0.05	<0.05	<0.05	<0.05	<0.05	<0.05	<0.05	0.14	<0.05	<0.05	<0.05	<0.05	<0.05	<0.05	<0.05
TA_R_	t	3.40	6.18	1.38	1.41	−4.50	−10.1	−0.28	0.32	−4.28	−1.64	0.036	0.68	−1.46	2.16	−2.81	−12.4
	p	<0.05	<0.05	0.24	0.23	<0.05	<0.05	0.79	0.77	<0.05	0.18	0.97	0.54	0.22	0.097	<0.05	<0.05
RF_R_	t	5.00	19.3	4.57	9.43	4.26	3.10	6.91	6.82	1.23	2.51	1.33	1.24	−1.31	3.39	4.73	9.11
	p	<0.05	<0.05	<0.05	<0.05	<0.05	<0.05	<0.05	<0.05	0.29	0.066	0.25	0.28	0.26	<0.05	<0.05	<0.05
BFL_R_	t	−2.13	−2.92	−1.71	1.03	0.56	−1.96	−2.67	−1.16	2.25	1.75	−6.71	−2.26	−0.79	−2.45	1.21	2.59
	p	0.10	<0.05	0.16	0.36	0.61	0.12	0.056	0.31	0.088	0.15	<0.05	0.087	0.47	0.071	0.29	0.061
**OFF centers on the KNEE torque plane**.																	
MG_L_	t	13.0	8.43	0.39	0.73	9.30	13.7	2.67	5.05	7.99	5.65	9.30	9.39	5.46	15.4	4.01	5.12
	p	<0.05	<0.05	0.71	0.51	<0.05	<0.05	0.056	<0.05	<0.05	<0.05	<0.05	<0.05	<0.05	<0.05	<0.05	<0.05
LG_L_	t	5.33	2.68	0.95	3.33	4.42	10.1	3.16	5.32	3.02	33.6	3.23	7.73	2.26	9.74	3.51	4.26
	p	<0.05	0.055	0.39	<0.05	<0.05	<0.05	<0.05	<0.05	<0.05	<0.05	<0.05	<0.05	0.086	<0.05	<0.05	<0.05
SOL_L_	t	6.79	20.1	3.32	4.05	3.59	7.98	1.91	5.41	1.59	15.6	7.08	7.33	6.69	12.4	3.03	5.21
	p	<0.05	<0.05	<0.05	<0.05	<0.05	<0.05	0.13	<0.05	0.19	<0.05	<0.05	<0.05	<0.05	<0.05	<0.05	<0.05
TA_L_	t	−0.85	−3.88	−2.06	−2.74	1.10	0.31	2.35	1.95	1.79	2.70	0.52	−0.075	−0.94	3.45	5.25	6.00
	p	0.44	<0.05	0.11	0.052	0.33	0.77	0.078	0.12	0.15	0.054	0.63	0.94	0.40	<0.05	<0.05	<0.05
RF_L_	t	−0.81	−2.69	−4.65	−7.41	−5.32	−8.04	−0.081	−0.99	−2.33	0.61	−1.14	−1.21	−0.59	−5.57	−3.62	−5.94
	p	0.47	0.055	<0.05	<0.05	<0.05	<0.05	0.94	0.38	0.080	0.57	0.32	0.29	0.59	<0.05	<0.05	<0.05
BFL_L_	t	−0.53	−1.61	3.44	0.83	−0.79	−0.46	2.66	3.22	11.3	6.83	5.62	5.78	−0.77	0.48	−0.51	−4.45
	p	0.62	0.18	<0.05	0.45	0.47	0.67	0.056	<0.05	<0.05	<0.05	<0.05	<0.05	0.48	0.66	0.63	<0.05
MG_R_	t	4.81	10.3	2.83	9.31	6.91	10.2	9.20	8.74	7.66	32.7	6.90	14.8	4.65	7.74	2.25	11.4
	p	<0.05	<0.05	<0.05	<0.05	<0.05	<0.05	<0.05	<0.05	<0.05	<0.05	<0.05	<0.05	<0.05	<0.05	0.088	<0.05
LG_R_	t	5.29	5.92	4.42	9.75	5.46	6.89	11.2	18.0	2.99	5.98	3.81	3.36	2.77	10.5	2.43	10.7
	p	<0.05	<0.05	<0.05	<0.05	<0.05	<0.05	<0.05	<0.05	<0.05	<0.05	<0.05	<0.05	0.050	<0.05	0.072	<0.05
SOL_R_	t	7.21	8.12	4.00	8.60	11.4	5.47	8.84	10.9	3.63	9.03	7.41	5.22	5.63	7.43	3.74	11.1
	p	<0.05	<0.05	<0.05	<0.05	<0.05	<0.05	<0.05	<0.05	<0.05	<0.05	<0.05	<0.05	<0.05	<0.05	<0.05	<0.05
TA_R_	t	−3.16	−5.16	−1.25	−1.51	3.86	5.24	1.18	0.52	3.85	1.87	0.32	−0.43	0.47	−2.45	2.69	9.83
	p	<0.05	<0.05	0.28	0.21	<0.05	<0.05	0.30	0.63	<0.05	0.13	0.77	0.69	0.66	0.071	0.054	<0.05
RF_R_	t	−5.93	−18.3	−2.89	−9.85	−4.25	−2.91	−5.93	−5.74	−0.71	−4.69	−1.11	−0.91	−0.23	−4.19	−3.71	−5.81
	p	<0.05	<0.05	<0.05	<0.05	<0.05	<0.05	<0.05	<0.05	0.52	<0.05	0.33	0.41	0.83	<0.05	<0.05	<0.05
BFL_R_	t	2.30	3.45	0.82	−1.20	−0.77	1.47	2.88	1.38	−1.19	−1.73	3.49	4.54	1.11	2.56	−1.42	−2.73
	p	0.083	<0.05	0.46	0.30	0.48	0.21	<0.05	0.24	0.30	0.16	<0.05	<0.05	0.33	0.063	0.23	0.052
**ON centers on the HIP torque plane**.																	
MG_L_	t	−11.9	−22.5	−1.41	−1.48	−5.50	−6.14	−2.93	−6.51	−6.63	−18.5	−12.3	−6.83	−15.0	−9.97	−3.30	−4.20
	p	<0.05	<0.05	0.23	0.21	<0.05	<0.05	<0.05	<0.05	<0.05	<0.05	<0.05	<0.05	<0.05	<0.05	<0.05	<0.05
LG_L_	t	−3.86	−2.82	−0.80	−3.61	−4.39	−9.76	−2.16	−5.09	−2.32	−7.78	−3.74	−11.8	−2.48	−10.1	−3.09	−4.09
	p	<0.05	<0.05	0.47	<0.05	<0.05	<0.05	0.096	<0.05	0.081	<0.05	<0.05	<0.05	0.068	<0.05	<0.05	<0.05
SOL_L_	t	−4.75	−9.35	−4.08	−6.86	−3.79	−9.78	−2.31	−6.92	−1.29	−4.59	−6.83	−9.59	−8.33	−14.6	−3.46	−4.98
	p	<0.05	<0.05	<0.05	<0.05	<0.05	<0.05	0.082	<0.05	0.27	<0.05	<0.05	<0.05	<0.05	<0.05	<0.05	<0.05
TA_L_	t	1.57	3.94	2.24	3.37	−0.34	−0.54	−2.25	−1.53	−1.94	−2.30	−0.28	−0.34	−1.67	−2.88	−4.25	−5.99
	p	0.19	<0.05	0.089	<0.05	0.75	0.62	0.088	0.20	0.12	0.083	0.79	0.75	0.17	<0.05	<0.05	<0.05
RF_L_	t	1.24	1.78	5.75	8.29	6.72	10.2	0.27	1.21	2.47	−0.37	1.61	1.66	0.19	0.96	4.63	11.5
	p	0.28	0.15	<0.05	<0.05	<0.05	<0.05	0.80	0.29	0.069	0.73	0.18	0.17	0.86	0.39	<0.05	<0.05
BFL_L_	t	0.87	1.03	−2.72	−1.39	0.63	−0.035	−1.93	−7.49	−12.6	−17.4	−4.18	−5.45	−2.59	−0.44	0.25	5.89
	p	0.43	0.36	0.053	0.23	0.56	0.97	0.13	<0.05	<0.05	<0.05	<0.05	<0.05	0.061	0.68	0.81	<0.05
MG_R_	t	−8.48	−4.57	−2.72	−8.38	−28.2	−5.86	−4.95	−7.51	−6.87	−9.60	−7.92	−5.82	−7.71	−9.06	−2.34	−9.74
	p	<0.05	<0.05	0.053	<0.05	<0.05	<0.05	<0.05	<0.05	<0.05	<0.05	<0.05	<0.05	<0.05	<0.05	0.079	<0.05
LG_R_	t	−5.01	−3.45	−3.87	−12.3	−7.03	−8.69	−6.44	−14.9	−2.50	−15.5	−6.81	−5.08	−2.79	−17.3	−2.55	−7.52
	p	<0.05	<0.05	<0.05	<0.05	<0.05	<0.05	<0.05	<0.05	0.067	<0.05	<0.05	<0.05	<0.05	<0.05	0.063	<0.05
SOL_R_	t	−10.2	−6.75	−4.64	−9.57	−11.8	−5.32	−5.81	−8.03	−2.08	−14.6	−15.1	−5.53	−3.77	−13.2	−3.92	−10.0
	p	<0.05	<0.05	<0.05	<0.05	<0.05	<0.05	<0.05	<0.05	0.11	<0.05	<0.05	<0.05	<0.05	<0.05	<0.05	<0.05
TA_R_	t	3.43	7.24	1.36	1.86	−3.50	−6.45	−0.26	−0.046	−4.34	−1.58	0.088	0.81	−1.77	0.81	−2.85	−27.5
	p	<0.05	<0.05	0.24	0.14	<0.05	<0.05	0.81	0.97	<0.05	0.19	0.93	0.46	0.15	0.46	<0.05	<0.05
RF_R_	t	5.49	14.6	4.69	9.08	4.60	2.96	7.15	6.38	1.28	2.54	1.30	1.40	−1.21	2.48	5.01	7.61
	p	<0.05	<0.05	<0.05	<0.05	<0.05	<0.05	<0.05	<0.05	0.27	0.064	0.26	0.24	0.29	0.068	<0.05	<0.05
BFL_R_	t	−2.02	−1.82	−1.66	1.09	0.54	−1.97	−2.67	−1.04	2.36	1.66	−6.45	−2.45	−1.20	−1.43	1.32	3.74
	p	0.11	0.14	0.17	0.34	0.62	0.12	0.056	0.36	0.078	0.17	<0.05	0.070	0.30	0.23	0.26	<0.05
**OFF centers on the HIP torque plane**.																	
MG_L_	t	12.4	11.4	0.35	0.67	9.45	12.2	2.67	5.36	7.12	5.20	9.05	10.0	5.89	17.5	4.07	4.04
	p	<0.05	<0.05	0.74	0.54	<0.05	<0.05	0.056	<0.05	<0.05	<0.05	<0.05	<0.05	<0.05	<0.05	<0.05	<0.05
LG_L_	t	5.15	3.63	0.92	3.30	4.42	8.79	3.13	5.02	3.07	8.52	3.08	8.16	2.86	11.0	3.51	3.86
	p	<0.05	<0.05	0.41	<0.05	<0.05	<0.05	<0.05	<0.05	<0.05	<0.05	<0.05	<0.05	<0.05	<0.05	<0.05	<0.05
SOL_L_	t	6.63	15.7	3.34	3.80	3.77	9.09	1.96	5.60	1.62	13.5	7.00	8.05	6.80	9.97	3.15	4.97
	p	<0.05	<0.05	<0.05	<0.05	<0.05	<0.05	0.12	<0.05	0.18	<0.05	<0.05	<0.05	<0.05	<0.05	<0.05	<0.05
TA_L_	t	−0.91	−3.95	−2.10	−2.71	0.97	0.48	2.35	2.44	1.92	3.09	0.54	0.17	−0.79	2.30	4.78	4.64
	p	0.42	<0.05	0.10	0.054	0.39	0.66	0.079	0.071	0.13	<0.05	0.62	0.87	0.47	0.083	<0.05	<0.05
RF_L_	t	−0.87	−3.05	−4.57	−9.05	−5.61	−8.06	−0.096	−1.14	−2.30	0.53	−1.08	−1.22	−0.63	−1.50	−3.70	−6.14
	p	0.43	<0.05	<0.05	<0.05	<0.05	<0.05	0.93	0.32	0.083	0.62	0.34	0.29	0.56	0.21	<0.05	<0.05
BFL_L_	t	−0.58	−1.62	3.27	0.63	−0.82	−0.37	2.73	3.11	10.5	7.18	5.76	5.40	−0.66	0.21	−0.62	−4.41
	p	0.59	0.18	<0.05	0.56	0.46	0.73	0.053	<0.05	<0.05	<0.05	<0.05	<0.05	0.55	0.84	0.57	<0.05
MG_R_	t	4.89	10.6	2.87	8.63	7.26	9.94	9.59	7.56	8.41	19.7	5.96	12.9	4.89	6.14	2.29	10.7
	p	<0.05	<0.05	<0.05	<0.05	<0.05	<0.05	<0.05	<0.05	<0.05	<0.05	<0.05	<0.05	<0.05	<0.05	0.084	<0.05
LG_R_	t	5.25	5.31	4.54	9.95	5.44	7.21	11.4	11.6	3.04	6.06	3.82	3.33	3.01	8.79	2.52	11.9
	p	<0.05	<0.05	<0.05	<0.05	<0.05	<0.05	<0.05	<0.05	<0.05	<0.05	<0.05	<0.05	<0.05	<0.05	0.065	<0.05
SOL_R_	t	7.36	6.28	4.12	7.66	10.4	5.24	9.15	16.7	3.54	6.39	7.45	4.36	5.88	8.20	3.69	9.59
	p	<0.05	<0.05	<0.05	<0.05	<0.05	<0.05	<0.05	<0.05	<0.05	<0.05	<0.05	<0.05	<0.05	<0.05	<0.05	<0.05
TA_R_	t	−3.15	−4.29	−1.26	−2.34	3.49	5.33	1.16	0.63	3.91	2.01	0.22	−0.78	0.82	−1.46	2.69	19.6
	p	<0.05	<0.05	0.28	0.080	<0.05	<0.05	0.31	0.56	<0.05	0.11	0.84	0.48	0.46	0.22	0.054	<0.05
RF_R_	t	−6.53	−10.4	−3.00	−10.4	−4.62	−2.76	−6.20	−5.13	−0.80	−4.33	−1.10	−0.83	−0.16	−3.27	−3.84	−6.02
	p	<0.05	<0.05	<0.05	<0.05	<0.05	0.051	<0.05	<0.05	0.47	<0.05	0.33	0.45	0.88	<0.05	<0.05	<0.05
BFL_R_	t	2.25	1.59	0.76	−1.25	−0.76	1.46	2.84	1.35	−1.33	−1.68	3.35	3.78	1.43	3.44	−1.54	−3.31
	p	0.088	0.19	0.49	0.28	0.49	0.22	<0.05	0.25	0.25	0.17	<0.05	<0.05	0.23	<0.05	0.20	<0.05

One-sample t-test was conducted for investigating whether on/off centers substantially departed from the x- and y- axes (joint angle (θ) and velocity (ω) axes in the phase plane, and joint torque (T) and torque velocity (dT/dt) in the torque plane, respectively) for five trials of each participant and each muscle. Abbreviations of “_L” and “_R” after the name of muscles represent left and right, respectively. Statistical values of *t* and *p* are shown for joint anlge and velocity (on phase planes) and joint torque and torque velocity (on torque planes) separately. We bold/underline the cell where there was a significant difference (p < 0.05).

The centers of on/off areas on the torque planes showed that phasic muscle activation and inactivation were associated with joint torque generation in the direction of anatomical action and in the opposite direction, respectively (Fig. 2). One of the most important results in this study is that muscle inactivation itself was also associated with the torque generation in the anatomically opposite direction and that the individual differences and laterality of the on/off area distribution were relatively small in the torque planes, in contrast with those in the phase planes. These results indicate that the function of intermittent muscle activity is to generate joint torque precisely in the direction of action and that such on/off trigger is modulated based on mechanical properties of the body or afferent/efferent transmission time lag. Intermittent feedback control strategy for human bipedal standing has been discussed at the kinematic level; however, information on muscle activities related to this has not been obtained. This study deepens our understanding of the intermittent control model to the musculoskeletal level and supports its validity. We also observed on/off area separation in anatomically irrelevant torque planes (Fig. 2, gray background figures). Further analysis of skeletal fluctuations should clarify the contribution of intermittent muscle activity to reciprocal interaction between multiple body segments.

### On/off switching of intermittent muscle activity

It has been suggested that the only sensory information during quiet standing is proprioceptive information^43, 44^. Proprioceptive information via mechanoreceptors is encoded to joint angle and angular velocity, which would allow the CNS to calculate SI during the inverse dynamic transformation together with anatomical or anthropometry information such as segment length, weight, and inertia. Our results indicated that there exists the structure in which all of above mentioned information is integrated and SI is calculated inside if the inverse model, which sense the condition of switching and generate descending motor command. Also, it could be possible that the switching condition depends on the second or higher derivative of SI, but it would be better to be based on lower derivative of SI as much as possible because higher derivative requires larger sampling points, which increases the burden of calculation and time lag.

Our event-driven intermittent feedback control model for human bipedal standing assumes that on/off switching of active control via the CNS is triggered based on the dynamics towards the equilibrium along with stable manifolds of the dynamical system^23–26, 31, 37, 38^. Thus, we hypothesized that the movement of the state point in the postural control system was dominated by the dynamics of the unstable equilibrium of the saddle type during the off-period and that it moved against the current of such dynamics during the on-period to return to the vicinity of the stable manifolds or the equilibrium. The comparison of the $\tilde{S}I$, which is the on/off switching principle for the simulation with intermittent feedback control theory, between on-periods and off-periods for experimental data showed no statistically significant differences (Fig. 3 right-top). On the other hand, the derivative values of $\tilde{S}I$ ($\tilde{S}I$) during the off-periods were significantly larger than those during on-periods for some muscles (Fig. 3 right-bottom). This inconsistency between the model principle and actual phasic muscle on/off switching may lead us to modify the intermittent feedback control strategy; therefore, we implemented a computer simulation with on/off switching based on $\tilde{S}I$.

**Figure 6:**
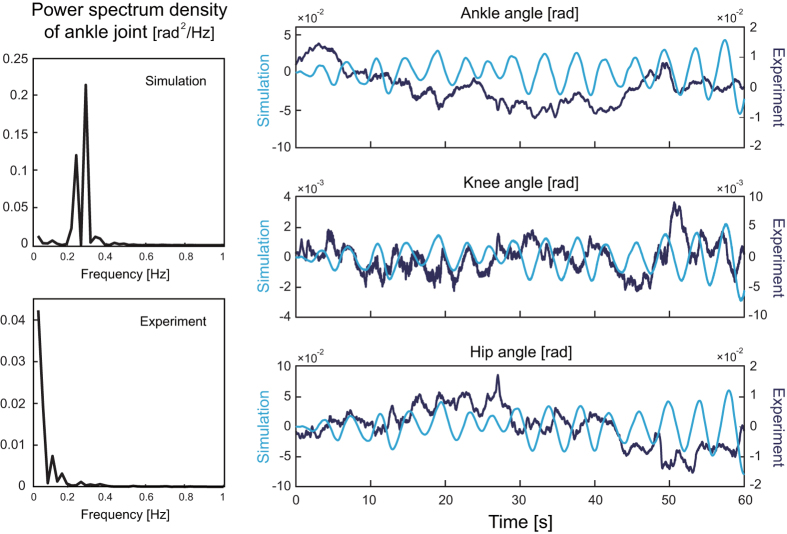
Power spectrum density of ankle angle (left) and joint fluctuations (right) for simulation and experimental data. Time series of joint angle for 60 s are shown in light blue lines for the simulation data and dark blue lines for the experimental data.

Figure 6 shows a comparison of power spectrum density of the ankle and joint fluctuations between simulation and experimental data. Although it is necessary to precisely tune the parameters so that the simulated data is similar to experimental data, we were not able to obtain the simulation data that resembles the actual body sway as much as we have previously demonstrated with a quadruple inverted pendulum^31^. We empirically know through our previous simulation process^31^ that the viscosity parameter ‘*B*’ (especially at the hip joint) affects the stability of the pendulum and makes the sway of the pendulum very similar to that of actual human body sway, but the tuning of viscosity parameter with a triple inverted pendulum did not work as much as it did with a quadruple pendulum. This is a great bottleneck of this study, but we believe that our simulation output still roughly resembles the actual body sway during quiet standing considering that it is extremely difficult to even keep standing the pendulum. Of course such bottleneck lowers the reliability of switching strategy based on the derivative of SI, but we found that the derivative of SI is differ between on- and off- periods for some muscles with a physiologically reasonable time lag (Fig. 4), indicating the possibility that event-driven on/off switching based on the system stability occurs in our postural control system. Further precision improvement of simulation is necessary for validating the switching strategy of muscle activation.

In this study, we assume that both *SI* and *SI* derivative are low when the system is unstable. The problem of the controller for human control is that the controller cannot produce the output large enough to stabilize the body by itself due to time-delayed instability. Thus, we assume that the controller outputs the motor command in an event-driven intermittent manner to compensate for time-delayed instability^29^. Thus, we assume that the system should be active so that the stability is increasing and decreasing *SI* would be a trigger of the activation. Of course the combination of *SI* and *SI* derivative would be better as a stabilization strategy, however, we investigated the possibility of SI and SI derivative separately to be trigger information of switching mechanism as a first step. In the comparison of $\tilde{S}I$ between on- and off- periods, some muscles (RF of participants 6 and 8, and MG of participant 7) showed significantly larger $\tilde{S}I$ during off-periods than that during on-periods with a physiologically reasonable time delay ranging from 100 to 200 ms (Fig. 4). This implies that these muscles’ on/off switching could be based on the ratio of stable/unstable components defined in the phase space of the triple inverted pendulum at around 100 to 200 ms before the on/off switching. Although it is impossible to precisely estimate time delay from the results in Fig. 4 because it was closed-loop condition in this study, the time delay used in Fig. 4 is in line with the time lag in the postural control feedback loop, which includes transmission from the somatosensory system to the brain lasting about 35 to 40 ms^45^, neural transmission from the brain to muscles lasting about 27 to 37 ms^46, 47^, electromechanical delay^48–51^, and a psychological refractory period^52–54^. Thus, these results partially validate our intermittent feedback control theory^31^, which involves intermittent actuation of the skeletal body based on the dynamics along with stable and unstable manifolds. However, for some of the other muscles (BFL of participants 3 and 5), the results contradicted the theory; the $\tilde{S}I$ during off-periods was significantly smaller than that of on-periods. This might have been due to the precision of simulation or the difference in the definition of stability between the kinematic and kinetic levels; even though the state point is moving along the stable manifolds (increase of kinematic stability), if the dynamics is going in the opposite direction to the anatomical action, the muscle may have to be activated. Thus, our results also imply that the dynamics along with stable manifold could be a trigger of phasic muscle activation when it occurs inside the space opposite to its anatomical action direction, which is shown as the on/off area in the phase planes in Fig. 1 and Table 2.

## Conclusion

The new insights of this study are that 1) muscles are likely to activate in an event-driven manner during quiet standing and a possible metric for on/off switching is SI dot, and 2) our methodology of EMG processing could allows us to extract such event-driven intermittent muscle activities. In this study, we demonstrated the direct relationship among joint fluctuation, muscle activities, and torque output during quiet standing by determining intermittent muscle activation and inactivation using EMG signals. We found it possible to extract intermittent on/off components of muscle activities by applying filtering technique to EMG signals. We statistically demonstrated the relationship between muscle on/off activity and joint fluctuation (on phase planes) and torque output (torque planes), which would help provide a necessary condition of muscle activation pattern for generating joint fluctuations and torque output during standing. Also, the similarity between experimental and simulated data (Fig. 6) and statistical difference in stability between on and off periods with physiologically reasonable time lag would provide an evidence for event-driven intermittent muscle activity for postural control. Our results also suggest that intermittent muscle activation/inactivation may be based on the rate of change in the stability component in the phase space, leading to joint actuation via torque generation in the direction of anatomical action, which enables us to maintain an upright posture.

## Materials and Methods

### Ethics statement

All procedures used in this study were in accordance with the Declaration of Helsinki and were approved by the Ethics Committee of the Graduate School of Human and Environmental Studies at Kyoto University. The approval was based on an appropriate risk/benefit ratio and a study design wherein the risks were minimized. All procedures were conducted in accordance with the approved protocol. The individuals participating in this study has given written informed consent to participate in this study and to publish these case details. Informed consent continued throughout the study via a dialog between the researcher and participants.

### Experimental protocol and measurement

Eight healthy males (age, 22.3 ± 1.7 years; height, 170.9 ± 7.9 cm; weight, 63.5 ± 5.7 kg) participated in this study. None of the participants had a significant medical history or signs of gait, postural, or neurological disorders, and none had vision problems. The participants were instructed to stand quietly with their eyes open and to look at a fixed point on a plain wall about 1.5 m ahead of them. They stood on a force platform (EFP–A–1.5kNSA13B, Kyowa, Tokyo, Japan) and kept standing for 120 s. We collected data from five trials for each participant with sufficient rest between trials (lasting a few minutes). The participants held their arms comfortably by their sides with their feet together. One splint (600 g) was strapped to the back of each participant at the forehead, chest, and pelvis to ensure a correct triple inverted pendulum model approximation of quiet standing by allowing motions to occur around the ankle, knee, and hip joints. When there was a gap between the head and splint, we put a light cushion (200 g) between them and filled the space so that the participants feel as if they were standing naturally as much as possible. Although this restriction could disturb the natural characteristics of quiet standing, we used it to measure the motion of a three-segmented body without ambiguity.

Joint motion data were obtained with a three-dimensional (3D) optical motion capture system (OptiTrack V100:R2; NaturalPoint, Corvallis, OR) composed of 12 infrared cameras in a semicircular arrangement. Spherical reflective markers, 13 mm in diameter, were affixed to the lateral side of the fifth metatarsophalangeal (MP), ankle (lateral malleolus), knee (lateral condyle of femur), hip (greater trochanter), anterior superior iliac spine (ASIS), and shoulder (acromion) on both sides of each participant’s body. We also placed one reflective marker on the reference point of the force platform to make the coordinate system of the platform in accordance with that of the motion capture system. The kinematic signals were sampled at a rate of 100 Hz and stored on the hard disk of a personal computer for subsequent off-line analysis.

Surface electromyography (EMG) from the skin surface over the rectus femoris (RF), long head of the biceps femoris (BFL), medial gastrocnemius (MG), lateral gastrocnemius (LG), soleus (SOL), and tibialis anterior (TA) was recorded from both legs with Ag-AgCl electrodes of 5 mm and an interelectrode distance of 20 mm. To minimize the cross talk between adjacent muscles, we first ascertained the location of the abdomen of each muscle by using an ultrasound method for attaching electrodes. After careful shaving and abrasion of the skin, the electrodes were placed over the abdomen of muscles. The reference electrode for EMG was placed over the lateral malleolus of the left leg. The electrodes were connected to a preamplifier and a differential amplifier with a bandwidth of 5-1000 Hz (MEG–6116 M, Nihon–kohden, Tokyo, Japan). All EMG signals were stored with a sampling frequency of 2 kHz on the hard disk of a personal computer using a 16-bit analog-to-digital converter (PowerLab/16SP, ADInstruments, Sydney, Australia). Data processing was performed with Matlab (MathWorks, USA).

### Data analysis

Time series of marker position coordinate data and the displacement of center of pressure (CoP) from the MP joint were passed through a second-order Butterworth low-pass filter with a cut-off frequency of 20 Hz (*filtfilt* function in the Matlab signal processing toolbox). The standing body was modeled as a triple inverted pendulum consisting of three rigid segments, namely, shank, thigh, and head-arm-trunk (HAT). The coordinates of the MP, ankle, knee, and top end of HAT were determined by the middle points of markers affixed on both sides of the MP, ankle, knee, and shoulder, respectively. The coordinates of the hip were calculated by using the marker location data of the greater trochanter and ASIS^55^. We then computed the segment lengths (MP–ankle, ankle–knee, knee–hip, and HAT) and joint angles of the ankle, knee, and hip in the sagittal plane. The angular velocities and angular accelerations were computed by numerically differentiating the angular displacement data with a three-point central difference formula^56^. Joint torques of the ankle, knee, and hip were calculated by inverse dynamics using vertical grand reaction force. The calculation procedures for joint angles and torques are presented in Supplementary Material A. Joint angles and torques were defined as positive in extension (Fig. A1).

**Figure 7:**
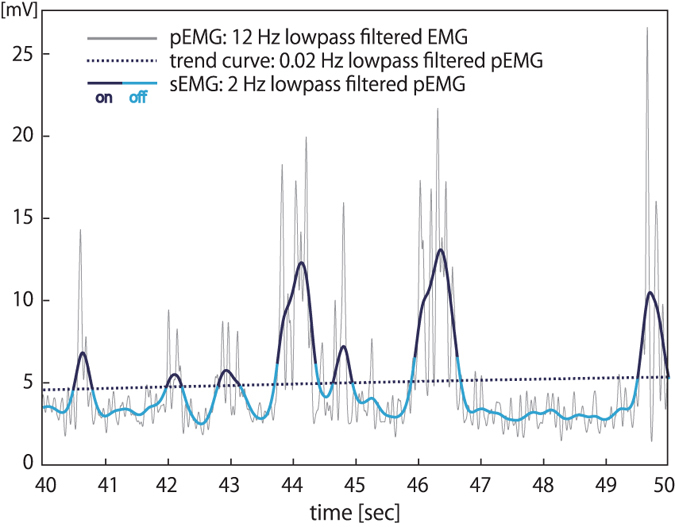
An example of on/off detection for 10 s of EMG data. Gray plot represents 12 Hz low-pass-filtered EMG (pEMG). Navy-dashed gradual curve and dark-light blue curve represent the trend curve (0.02-Hz low-pass-filitered pEMG) and sEMG (2 Hz lowpass filtered pEMG), respectively. We consider the trend curve to represent continuous and tonic muscle activity without active postural control via the CNS, and sEMG to represent a mixture of continuous and intermittent components.

All EMG signals were first numerically rectified and processed by the second-order Butterworth low-pass filter with a cut-off frequency of 12 Hz (pEMG: processed EMG). We determined intermittent muscle activation and inactivation (on-period and off-period) from EMG data by using two low-pass filtered EMG signals based on the work of Nomura *et al*.^35^ as follows. A second order Butterworth low-pass filter with a cut-off frequency of 0.02 Hz was applied to all pEMG signals to obtain trend curves, which represent tonic muscle activity components. We used this cut-off frequency of 0.02 because the cross-correlation between the center of mass (CoM) and low-pass filtered EMG of SOL during quiet standing was the highest when its cut-off frequency ranged between 0.02 and 0.06 Hz^35^ and our findings in this study were not affected by this cut-off frequency when we changed it to 0.01 or 0.07 Hz. We have also tried a constant threshold based on RMS amplitudes for on/off detection and we could not found clear relationship of on/off center distributions with either joint oscillation or joint torque, suggesting that a linear threshold would be useless for detecting muscle activation/inactivation associated with postural control. We also obtained smoothed pEMG signals (sEMG: smoothed EMG) by applying the second order Butterworth low-pass filter with a cut-off frequency of 2 Hz, assuming that the trend curve subtracted from sEMG represents intermittent muscle activation due to postural control via the CNS. If the sEMG was above the trend curve for a certain period, we considered that the muscle activity was high in that period. In this way, we obtained intervals in which the muscle activity was high. Then, for each interval with high muscle activity, we further computed the maximum value of the sEMG. If half of the maximum value was greater than the trend curve, it was defined as the threshold of the interval. Otherwise, the trend curve itself was defined as the threshold of the interval. We determined the threshold curve by performing this procedure for every interval with high muscle activity. An example of on/off periods determined by a single pEMG signal is shown in Fig. 7. The variability of off- and on- durations vary from 0.5 to 2 sec, indicating that clock-driven postural control is not plausible at least.

We then investigated the relationship between intermittent muscle activities and joint oscillations by dividing the dynamics in the phase planes (that is, joint angle vs. angular velocity relationship of the ankle, knee, and hip) into on- and off- period areas for each of the 12 muscles. Each on/off area was fitted into a two-component mixed Gaussian distribution for every muscle using a Matlab function: $obj\,=gmdistribution.fit([\theta ,\dot{\theta }],\,2)$. Then, the centers of each on/off area were calculated from the input matrix of means mu (*obj.mu*), input array of covariance (*obj.Sigma*), and input vector of mixing proportions (*obj.PComponents*). In the same way, we examined the distribution of the on/off area in the torque plane (torque vs. torque derivative relationship of the ankle, knee, and hip) for each muscle in order to investigate the relationship between intermittent muscle activity and joint torque output. On/off centers on the phase planes and torque planes are shown as a normalized data; each center was normalized by the maximum values of each trial’s angular position (x-axis) and angular velocity (y-axis) for the phase planes and of each trial’s joint torque (x-axis) and the derivative of joint torque (y-axis) for the torque planes. Coordinates of on/off centers for some trials were more than 1, which was due to the fact that the Gaussian distribution is broader than the actual on/off area and its centers located outside of the actual area.

We further investigated whether intermittent muscle activity originated from a feedback loop via the CNS or from reflex loops. In the concept of our event-driven intermittent feedback control strategy^31, 37, 38^, joint torque is intermittently triggered based on the distance between state point (which consists of joint angle and velocity) and stable/unstable manifolds approximately 200 ms ago, which assumes a time delay for neural processing and electromechanical coupling. The motion equation of the triple inverted pendulum as a model of human bipedal standing can be linearized as follows because joint angles and velocities are small during quiet standing, allowing us to neglect the second- and higher- order terms:

1 $$M\ddot{\theta }+G\theta =T$$

where θ is a joint angle vector, *M* the inertia matrix, Gθ the gravitational toppling torque vector, and T the joint torque vector. Eq. 1 can be expressed as the state space representation:

2 $$dy/dt=Ay$$

where *y* is a state variable consisting of three joint angles (θ) and three angular velocities (ω): *y(t) = (θ(t), ω(t))* ^*T*^ at time *t* (we consider only joint angle and angular velocity to be state variables in this study and joint torque to be the external control signal at the output of the neuromuscular system), and *A* is a state matrix of the off-period (without any active feedback control). Matrices *M* and *G* in eq. 1 determine state matrix *A*, and they were calculated by using Japanese anthropometric parameters shown in Table 3 ^57^. The definitions of these two matrices and the state space representation of the motion equation are described in Supplementary Material B1 and B2. The eigenvalues and eigenvectors of state matrix *A* determine the dynamics of the state point in the phase space. For our triple inverted pendulum model with human-like anthropometric parameters, five stable manifolds and one unstable manifold governed the pendulum without any control input. The mapping from phase space to eigenvector space is as follows:

3 $$x={V}^{-1}y$$

where *V* is a matrix that consists of eigenvectors of the state matrix *A*, and x is a vector of one unstable component (*x*_*1*_) and five stable components (*x*_*2*_, …, ×_*6*_).

**Table 3:** Anthropometric parameters of a triple inverted pendulum.

Body mass [kg]	for each subject
Segment length [m]	
Segment mass ratio [% body mass]	[10.2 22.0 67.8]
Segment center of mass ratio [%]	[40.6 47.5 49.3]
Gyration radius ratio [%]	[27.4 27.8 34.6]
Elastic component [K_a_, K_k_, K_h_,]	[0.8 0.5 0.5]*mgh
Viscosity component [B_a_, B_k_, B_h_,]	[4 10 10]

The three values in each row (except for body mass and segment length) are for shank, thigh, and HAT segments, from left to right. Segment center of mass ratio is with respect to the segment length from the upper end. Gyration radius is relative to frontal (mediolateral) axis and is presented as a percentage of each segment length.

In our intermittent feedback control theory^31, 37, 38^, we assume that on/off switching is triggered based on the following stability index (*SI*), which is calculated by the stable and unstable components in the eigenvector space of off-system (eq. (2)):

4 $$SI=\alpha \sqrt{{|{x}_{2}|}^{2}+\cdots +{|{x}_{6}|}^{2}}-|{x}_{1}|$$

where α is a fixed value of 1/30 based on our previous study^31^. When *SI* > 0, active control is turned off 200 ms later, which includes the time delay for sensory feedback, neural processing, and torque generation/actuation, because of a sufficient amount of stability. Otherwise, active control is turned on to actuate the pendulum. For experimental data, we calculated the stability index using the state matrix *A* in simulation:

5 $$\tilde{S}I=\alpha \sqrt{{|{\tilde{x}}_{2}|}^{2}+\cdots +{|{\tilde{x}}_{6}|}^{2}}-|{\tilde{x}}_{1}|$$

6 $$\tilde{x}={V}^{-1}\tilde{y}$$

where $\tilde{y}$ is an actual state variable consisting of joint angle and velocity calculated from the experimental data. For investigating the possibility that actual muscle activation and inactivation occur based on the stability of the system within a range of physiologically reasonable time lag, we then compared $\tilde{S}I$ between on-periods and off-periods for each muscle by using $\tilde{x}$ with a variety of time lags ranging from 100 to 200 ms; however, our results showed that there was no significant difference in the value of $\tilde{S}I$, but in the derivative value of $\tilde{S}I$ ($\tilde{S}I$) between on- and off- periods. Thus, we compared the first-order differentiation of $\tilde{S}I$ ($\tilde{S}I$) between on- and off- periods for each muscle for time lags from 100 to 200 ms. We address this issue in the Discussion section. We also performed a computer simulation using a new intermittent feedback control strategy based on our experimental results; on/off switching uses the derivative value of *SI* ($\dot{SI}$) as a reference. This is for checking whether this new control strategy is valid as a model of the system of human postural control. Therefore, the intermittent joint torque generation occurred as follows:

7 $$T=\{\begin{array}{c}K\theta +B\dot{\theta }+P{\theta }_{{\rm{\Delta }}}+D{\dot{\theta }}_{{\rm{\Delta }}}\,({\dot{SI}}_{{\rm{\Delta }}}\le 0;on\_period)\\ K\theta +B\dot{\theta }\,({\dot{SI}}_{{\rm{\Delta }}} > 0;off\_period)\end{array}$$

where *K* and *B* are passive elastic and damping coefficients, respectively, and *P* and *D* are active feedback gains. Subscript Δ means that the state variable includes a time delay: θ_Δ_ = θ(t − Δ). Values of the parameters K, B, P, and D are provided in Supplementary Material B3. Because we have Heaviside switching (eq. (7)) in our feedback control model, eq. (1) does not exhibit saddle point instability as a whole system. However, if we focus only on the system during off-period (eq. (2)), the system is a saddle type consisted of five stable manifolds and one unstable manifold and there exists an equilibrium point in the off-system. We assume that this saddle point of off-system could be incorporated in the internal model as a “ghost equilibrium point” in the process of inverse dynamic transformation inside of the CNS even during on-periods.

### Statistical analysis

For statistical investigation of the distribution of on/off centers in the phase and torque planes, we conducted one-sample t-test and checked whether on/off centers substantially departed from the x- and y- axes (joint angle and velocity axes in the phase plane, and joint torque and torque velocity in the torque plane, respectively) for five trials of each participant and each muscle. Significant levels of differences between five-sample data and zero were tested using *t-test* in the statistical toolbox of Matlab. A comparison of *SI* between on- and off- periods was also implemented using an independent *t*-test when a normal distribution could be assumed; otherwise, we used the Mann-Whitney U-test. Both tests were performed using the functions of *t-test* and *signrank* in the statistical toolbox of Matlab. The statistical significance threshold was set at *p* = 0.05.

## Electronic supplementary material

Supplementary material
