# Supplementary material for: Intermittent muscle activity in the feedback loop of postural control system during natural quiet standing
Source: Sci Rep. 2017 Sep 6;7:10631. doi: 10.1038/s41598-017-10015-8 (PMC5587544; doi:10.1038/s41598-017-10015-8)
Supplement: Supplementary file 1 — Supplementary material [file 41598_2017_10015_MOESM1_ESM.pdf]

Supplementary materials for:

Intermittent muscle activity in the feedback loop of postural control system during natural quiet standing

H. Tanabe, K. Fujii, M. Kouzaki

This PDF file includes:

A. Joint angle and torque calculation

B. Motion equation of the inverted pendulum

A. Joint angle and torque calculation

Definitions of angular displacements of the ankle, knee, and hip are shown in Figure A1. Ankle dorsiflexion, knee extension, and hip forward flexion were defined to be positive direction.

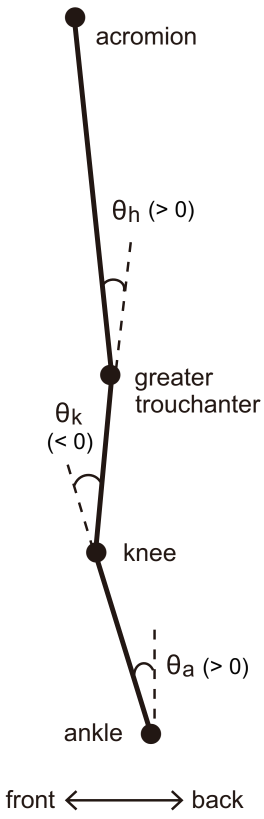

Figure A1: A triple inverted pendulum model. A model for a human quiet standing in the sagittal plane. Each of four links represent shank (ankle–knee), thigh (knee–hip), and head-and-trunk segments, from the bottom. Joint angles were defined as relative angles between adjacent joints except for joint angle of the MP being relative to the vertical line.

Joint torque was calculated by inverse dynamics. The x and y axes represent

anteroposterior and vertical directions, respectively. Due to the equilibrium of force at the lowest segment (i.e., the foot),

$$R_{ax} + F_x = 0$$

$$R_{ay} + F_y - m_f g = 0$$

where R_{ax} and R_{ay} represent the joint reaction force at the ankle in anteroposterior and vertical directions, respectively. F_x and F_y are the grand reaction force, where subscripts represent each direction of axis. The term $m_f g$ is gravitational force applied to the foot (i.e., m_f is a mass of the foot). The equilibrium of moment around the center of mass (COM) of foot segment leads to the following equation,

$$M_a + rR_{ay} - pF_y = 0$$

where M_a is the ankle moment in the planterflexion direction, and r and p are a horizontal distances between the ankle and foot COM and between foot COM and the center of pressure. Because we defined the ankle dorsiflexion direction to be positive, the ankle torque T_a is the opposite sign of M_a.

The motion equation of the upper segments (i.e. shank, thigh, and upper body) is expressed as follows:

$$m_i a_x = R_{xp} + R_{xd}$$

$$m_i a_y = R_{yp} + R_{yd} - m_i g$$

where m_i is a mass of ith segment, a_x and a_y are acceleration of ith segment's COM in anteroposterior and vertical directions, respectively, R_{xp} and R_{yp} are joint reaction forces

at the proximal end in the anteroposterior and vertical directions, and R_{xd} and R_{yd} are joint reaction forces at the distal end in the anteroposterior and vertical directions (i.e., joint reaction force at the ankle for the shank). These two equations lead the joint reaction force at the proximal end. Then, joint moment at the *i*th segment (equivalent to M_d in the following equation) can be derived from the following Euler's momentum equation:

$$I_i\ddot{\theta}_i = M_{pi} - M_{di} + R_{xp}r_i\cos\theta_i - R_{yp}r_i\sin\theta_i - R_{xd}(l_i - r_i)\cos\theta_i + R_{yd}(l_i - r_i)\sin\theta_i$$

where the subscript *i* represents the *i*th segment, I is the inertial moment, θ is the angular displacement, M_p and M_d are the joint moment in the backward direction (i.e., the ankle plantarflexion, knee flexion, and hip backward flexion) at the proximal and distal ends, respectively, r is a length between proximal end and segment COM, and l is a segment length. Because we defined the knee extension and hip forward flexion to be positive, the torque at the knee and hip is the opposite sign of M_p of shank and thigh segments, respectively.

B. Motion equation of the inverted pendulum

B.1 Model definition

$$M_{11} = I_1 + I_2 + I_3 + r_1^2m_1 + r_2^2m_2 + l_1^2m_2 + 2l_1m_2r_2$$

$$+r_3^2m_3 + l_1^2m_3 + l_2^2m_3 + 2l_1m_3l_2 + 2l_2m_3r_3 + 2l_1m_3r_3$$

$$M_{12} = I_2 + I_3 + r_2^2m_2 + l_1m_2r_2 + r_3^2m_3 + l_2^2m_3 + l_1m_3l_2 + 2l_2m_3r_3 + l_1m_3r_3$$

$$M_{13} = I_3 + r_3^2 m_3 + l_2 m_3 r_3 + l_1 m_3 r_3$$

$$M_{21} = I_2 + I_3 + r_2^2 m_2 + l_1 m_2 r_2 + r_3^2 m_3 + l_2^2 m_3 + l_1 m_3 l_2 + 2 l_2 m_3 r_3 + l_1 m_3 r_3$$

$$M_{22} = I_2 + I_3 + r_2^2 m_2 + r_3^2 m_3 + l_2^2 m_3 + 2 l_2 m_3 r_3$$

$$M_{23} = I_3 + r_3^2 m_3 + l_2 m_3 r_3$$

$$M_{31} = I_3 + r_3^2 m_3 + l_2 m_3 r_3 + l_1 m_3 r_3$$

$$M_{32} = I_3 + r_3^2 m_3 + l_2 m_3 r_3$$

$$M_{33} = I_3 + r_3^2 m_3$$

$$G_{11} = -g\left(r_1 m_1 + l_1 m_2 + l_1 m_3 + r_2 m_2 + l_2 m_3 + r_3 m_3\right)$$

$$G_{12} = -g\left(r_2 m_2 + l_2 m_3 + r_3 m_3\right)$$

$$G_{13} = -g m_3 r_3$$

$$G_{21} = -g\left(r_2 m_2 + l_2 m_3 + r_3 m_3\right)$$

$$G_{22} = -g\left(r_2 m_2 + l_2 m_3 + r_3 m_3\right)$$

$$G_{23} = -g m_3 r_3$$

$$G_{31} = -g m_3 r_3$$

$$G_{32} = -g m_3 r_3$$

$$G_{33} = -g m_3 r_3$$

where I_i, m_i, l_i, and r_i represent the ith segment's inertia moment around the distal end, the mass, the length, and the length between the distal end and center of mass, respectively.

B.2 First order differential equation

The passive joint torque in the motion equation (eq. 1) can be represented as follows:

$$T_{passive} = -\begin{bmatrix} diag(K) & diag(B) \end{bmatrix} \cdot x$$

where K and B are vectors of elastic and viscosity components, respectively, and x is the state variable vector consisted of three joint angles and three angular velocities. The expression *diag(v)* is a diagonal matrix composed by vector v.

Therefore, the motion equation (eq. 1) with no active torque can be written as a following six-dimensional ordinary first order differential equation:

$$\begin{bmatrix} E & O \\ O & M \end{bmatrix} \cdot \frac{dx}{dt} = \begin{bmatrix} O & E \\ -diag(K)+G & -diag(B) \end{bmatrix} \cdot x$$

where E is a 3-by-3 unit matrix. This elicits the coefficient matrix A in eq. 2 as follows:

$$A = \begin{bmatrix} E & O \\ O & M \end{bmatrix}^{-1} \begin{bmatrix} O & E \\ -diag(K)+G & -diag(B) \end{bmatrix}$$

B.3 PD gain and passive viscoelasticity

PD gains and passive viscoelasticity coefficients were set to be as follows based on our previous study (Tanabe et al. 2016) and other simulation studies (Loram and Lakie 2002; Casadio et al. 2005; Maurer and Peterka 2005; Bottaro et al.

2008; Asai et al. 2009; Suzuki et al. 2012):

$$[P_a \quad P_k \quad P_h] = [1.0 \quad 0.4 \quad 0.3] * mgh$$

$$[D_a \quad D_k \quad D_h] = [10 \quad 10 \quad 50]$$

$$[K_a \quad K_k \quad K_h] = [0.6 \quad 20 \quad 0.2] * mgh$$

$$[B_a \quad B_k \quad B_h] = [4 \quad 50 \quad 50]$$

We modified these values so that the joint oscillations of the pendulum shows the similar amplitude and variability as those of actual human quiet standing. It is important to set relatively larger value of D_h (> 50s) for preventing the model from falling down.
